# Dysbiosis of a microbiota–immune metasystem in critical illness is associated with nosocomial infections

**DOI:** 10.1038/s41591-023-02243-5

**Published:** 2023-03-09

**Authors:** Jared Schlechte, Amanda Z. Zucoloto, Ian-ling Yu, Christopher J. Doig, Mary J. Dunbar, Kathy D. McCoy, Braedon McDonald

**Affiliations:** 1 Department of Critical Care Medicine, Cumming School of Medicine, University of Calgary, Calgary, Alberta Canada; 2 Snyder Institute for Chronic Diseases, Cumming School of Medicine, University of Calgary, Calgary, Alberta Canada; 3 Department of Physiology and Pharmacology, Cumming School of Medicine, University of Calgary, Calgary, Alberta Canada; 4 Department of Community Health Sciences, Cumming School of Medicine, University of Calgary, Calgary, Alberta Canada; 5 Department of Pediatrics, Cumming School of Medicine, University of Calgary, Calgary, Alberta Canada; 6 Alberta Children’s Hospital Research Institute, Cumming School of Medicine, University of Calgary, Calgary, Alberta Canada

**Keywords:** Translational research, Infection, Microbial communities

## Abstract

Critically ill patients in intensive care units experience profound alterations of their gut microbiota that have been linked to a high risk of hospital-acquired (nosocomial) infections and adverse outcomes through unclear mechanisms. Abundant mouse and limited human data suggest that the gut microbiota can contribute to maintenance of systemic immune homeostasis, and that intestinal dysbiosis may lead to defects in immune defense against infections. Here we use integrated systems-level analyses of fecal microbiota dynamics in rectal swabs and single-cell profiling of systemic immune and inflammatory responses in a prospective longitudinal cohort study of critically ill patients to show that the gut microbiota and systemic immunity function as an integrated metasystem, where intestinal dysbiosis is coupled to impaired host defense and increased frequency of nosocomial infections. Longitudinal microbiota analysis by 16s rRNA gene sequencing of rectal swabs and single-cell profiling of blood using mass cytometry revealed that microbiota and immune dynamics during acute critical illness were highly interconnected and dominated by Enterobacteriaceae enrichment, dysregulated myeloid cell responses and amplified systemic inflammation, with a lesser impact on adaptive mechanisms of host defense. Intestinal Enterobacteriaceae enrichment was coupled with impaired innate antimicrobial effector responses, including hypofunctional and immature neutrophils and was associated with an increased risk of infections by various bacterial and fungal pathogens. Collectively, our findings suggest that dysbiosis of an interconnected metasystem between the gut microbiota and systemic immune response may drive impaired host defense and susceptibility to nosocomial infections in critical illness.

## Abstract (web summary)

Dysbiosis of the gut microbiome in critically ill patients, coupled with impaired innate immune responses, may lower the barrier to acquiring infectious pathogens in the hospital setting.

## Main

Critically ill patients requiring life-support interventions in intensive care units (ICUs) suffer very high rates of hospital-acquired (nosocomial) infections (20–50%) that contribute a markedly elevated risk of mortality^1,2^. Susceptibility to severe infections in critical illness has been linked to widespread impairment of innate and adaptive immunity and a breakdown of host defense mechanisms, as well as breaching of physical barriers by medical devices (intravascular catheters, endotracheal tubes and bladder catheters)^3–6^. In addition, nosocomial infections in the ICU are often caused by pathogens that are rarely associated with invasive disease in healthy individuals, consistent with a state of severely impaired host defense^1^; however, the mechanisms that induce and propagate immune dysfunction in critical illness are poorly understood.

Evidence from both mice and humans has revealed an important role for gut microbes in the maintenance of immune homeostasis and protective host defense in the gut as well as extra-intestinal, systemic compartments^7–9^. Critically ill patients harbor profound dysbiosis of their gut microbiota^10–14^ and this dysbiosis observed in ICU and other hospitalized patients has been associated with an increased risk of adverse outcomes, including infections through unclear mechanisms^11,14–16^. Colonization and overgrowth of pathobiont microbes may lead to translocation into the bloodstream as a direct mechanism linking gut dysbiosis to infections^15,17,18^; however, additional mechanisms likely contribute to the high rates of infections across multiple body sites in ICU patients that are caused by diverse pathogens beyond typical gut pathobionts^1^. In particular, alterations to the gut microbiota may render the host susceptible to infections through pathological crosstalk with the immune system, leading to impaired host defense.

In this study, we tested the hypothesis that susceptibility to nosocomial infections in critical illness is driven by pathological microbiota–immune interactions, in which gut microbiota dysbiosis triggers impaired systemic immunity and host defense. Using integrated systems-level analysis of gut microbiota dynamics and systemic immune function in 51 critically ill adults, we propose that the gut microbiota and systemic immune response behave as an integrated microbiota–immune metasystem, wherein dysbiosis characterized by progressive enrichment of Enterobacteriaceae in the gut microbiota leads to dysregulated innate immunity and impaired host defense and increased susceptibility to bacterial and fungal nosocomial infections.

## Results

### Pathological gut microbiota dynamics associate with nosocomial infections in critically ill patients

We conducted a prospective, longitudinal, integrated multi-omics analysis of the fecal microbiota, systemic cellular immune and inflammatory responses in 51 critically ill adults in medical, surgical, trauma and neurological ICUs in Calgary (Table 1). We enrolled patients who were adults, newly admitted to the ICU and expected to require continuous mechanical ventilation and intensive care for at least 72 h, as judged by the treating specialists. To avoid known confounders of microbiota ecology or systemic immune function, we excluded patients who were in hospital more than 48 h before ICU admission (during the current admission or any time in the previous 3 months), received systemic antibiotics in the 3 months before admission, were immunocompromised (congenital or acquired), had inflammatory bowel disease or gastrointestinal (GI) malignancy, had a discontinuous GI tract or moribund patients not expected to survive >72 h at the time of admission (Methods and Supplementary Table 1 provide additional details).

**Table 1:** Demographic, clinical and treatment characteristics of study participants

Characteristics	ICU patients (*n* = 51)	Healthy controls (*n* = 18)
Demographics		
Age (years), median (range)	61 (20–86)	32 (22–68)
Male sex, *n* (%)	31 (60.8)	8 (44.4)
Female sex, *n* (%)	20 (39.2)	10 (55.6)
Ethnicity, *n* (%)		
White	29 (56.9)	11 (61.1)
Asian	12 (23.5)	5 (27.8)
Black	3 (5.8)	1 (5.6)
Hispanic	1 (2.0)	0
Indigenous	6 (11.8)	1 (5.6)
Comorbidities		
Diabetes, *n* (%)	9 (17.6)	2 (11.1)
Cardiovascular disease, *n* (%)	15 (29.4)	0
Chronic lung disease, *n* (%)	10 (19.6)	2 (11.1)
Cirrhosis	0	0
Chronic kidney disease (on dialysis)	0	0
GERD, *n* (%)	11 (21.7)	2 (11.1)
Charlson index, median (range)	1 (0–8)	0 (0–1)
Admission diagnosis, *n* (%)		
Sepsis	24 (47.1)	NA
Trauma	12 (23.5)	NA
Neurological	10 (16.6)	NA
Medical (other)^a^	5 (9.8)	NA
Illness severity		
Admission SOFA score, median (range)	8.0 (2–16)	NA
Therapies, *n* (%)		
Invasive mechanical ventilation	51 (100)	NA
Antibiotics at ICU admission	28 (54.9)	NA
Enteral nutrition	51 (100)	NA
Parenteral nutrition	0 (0)	NA
Outcomes		
Nosocomial infection (to day 30), *n* (%)	28 (54.9)	NA
Duration of mechanical ventilation (days), median (range)	6 (1–24)	NA
Duration of ICU stay (days), median (range)	7 (2–31)	NA
Duration of hospitalization (days), median (range)	17 (4–207)	NA
Mortality (to day 30), *n* (%)	17 (33.3)	NA

^a^Medical (other) admission diagnoses include cardiac arrest, hemorrhagic shock and pulmonary embolism. NA, not available.

**Fig. 1:**
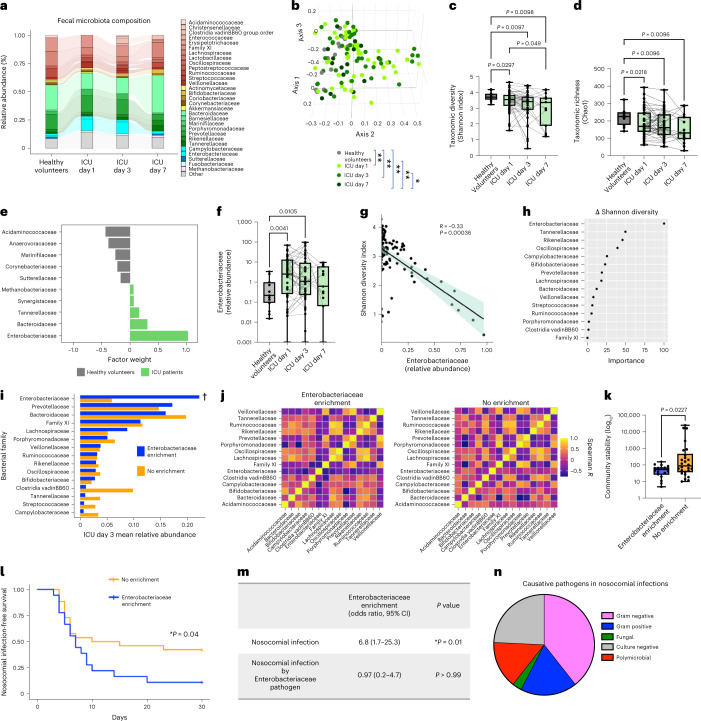
Intestinal dysbiosis with progressive Enterobacteriaceae enrichment in critical illness is associated with nosocomial infections. **a**, Taxonomic composition by relative abundance of bacterial families. **b**, Three-dimensional principal-coordinates analysis (Bray–Curtis dissimilarity distances, genus level) analyzed by PERMANOVA. **c**, Shannon index. **d**, Chao1 index in rectal swabs from critically ill patients on day 1 (*n* = 51) and again from survivors who remained in ICU on day 3 (*n* = 44) and day 7 (*n* = 15), compared to healthy volunteers (*n* = 15). Dots represent individual patients, central line indicates median, box shows interquartile range (IQR) and whiskers show range; analyzed by two-sided Kruskal–Wallis test (healthy versus ICU days) with pairwise comparisons of repeated measures across days using a mixed linear regression model with a post hoc Tukey’s test. **e**, MOFA of microbiota composition between healthy volunteers and ICU patients showing top ten taxonomic factors (families) and their relative contributions to explained microbiota variance (factor weight). **f**, Enterobacteriaceae relative abundance on days 1, 3 and 7 of ICU admission compared to healthy controls. Dots represent individual patients, central line shows median, box shows IQR and whiskers show range, analysis as per **c** and **d**. **g**, Correlation between Enterobacteriaceae relative abundance and Shannon index, analyzed using Spearman correlation test. Dots show individual patient samples, regression (line) and 95% confidence intervals (shaded area) are shown. **h**, Penalized ridge regression of the 15 most abundant bacterial families and their importance toward change in Shannon diversity from days 1–3 of ICU admission. **i**,**j**, Mean relative abundance († indicates *P*_adj _ < 0.1 by ANCOM-II differential abundance) (**i**) and correlation matrices (**j**) of the 15 most abundant bacterial families on ICU day 3. **k**, Longitudinal microbiota community stability index between patients with progressive Enterobacteriaceae enrichment (*n* = 18) or no enrichment (*n* = 26). Dots represent individual patients, central line shows the median, box shows IQR and whiskers show range; analyzed by two-sided Mann–Whitney *U*-test. **l**–**n**, The 30-d nosocomial infection-free survival analyzed by log-rank test (**l**), odds ratio of nosocomial infection caused by any pathogen or Enterobacteriaceae pathogen determined by two-sided Fisher’s exact test (**m**) and pathogens identified in nosocomial infections (**n**) (*n* = 30 infections in 28 patients). *P* values as shown in **b**; *P < 0.05, **P < 0.01.

**Extended Data Fig. 1:**
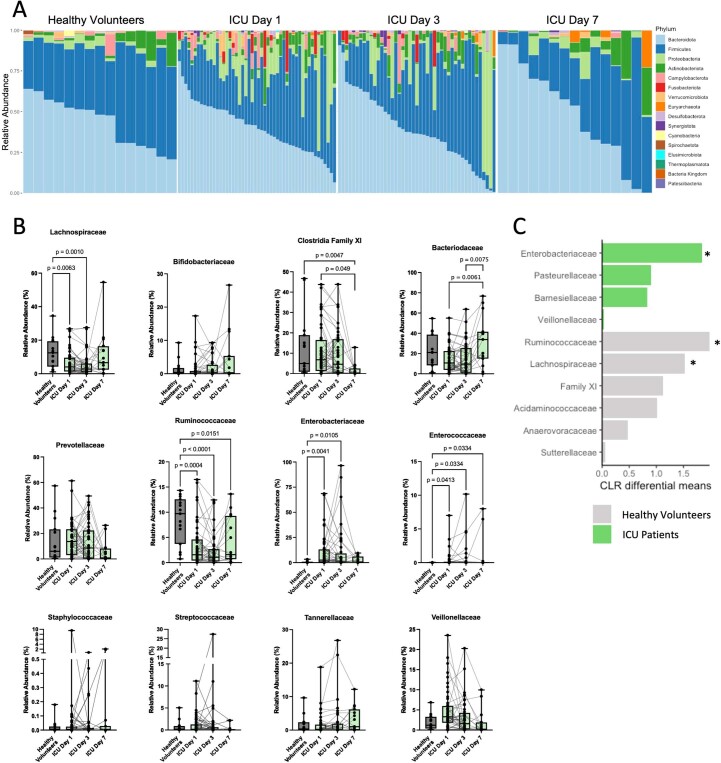
Fecal microbiota dynamics in critically ill patients. **(a)** Taxonomic composition (phylum level) and **(b)** analysis of the relative abundance of bacterial families in critically ill patients (N = 51) sampled on day 1 of admission (N = 51), and again from survivors who remined in ICU on day 3 (N = 44), and day 7 (N = 15), compared to healthy volunteer controls (N = 15). Dots are individual patients, central line is median, box shows IQR, whiskers show range. Statistical comparisons between healthy volunteers and ICU patients at each time point were performed using a Kruskal–Wallis test, while pairwise comparisons of repeated measures across ICU patient-days were performed using mixed linear regression model with post hoc Tukey’s tests to account for repeated measures and variable drop-out. P values as shown. **(c)** Differential abundance of bacterial families between ICU patients and healthy volunteers was determined using ANCOM-II with a mixed model to account for repeated measures (across sampling days) (*p-adj<0.1).

Fecal bacterial microbiota composition was analyzed at the time of ICU admission and then serially on days 3 and 7 of ICU admission using 16s rRNA gene amplicon sequencing (Fig. 1a). Owing to the need for precise timing of sample collection and unpredictable timing of bowel movements in critically ill patients, rectal swabs were utilized as previously described in multiple studies of gut microbiota in ICU patients^11,19–21^. From the time of admission, critically ill patients harbored evidence of gut dysbiosis compared to healthy volunteers, including reduced taxonomic diversity, richness and significant shifts in community β-diversity (Fig. 1a–d). Serial analysis of the microbiota over the first week of critical illness demonstrated progressive erosion of biodiversity, taxonomic richness and compositional shifts (Fig. 1a–d, Extended Data Fig. 1 and Supplementary Tables 2 and 3). Consistent with previous reports^10–12^, the shifts in fecal microbial communities in critical illness were characterized by a loss of commensal anaerobic fermenters (Ruminococcaceaea and Lachnospiraceae) and emergence of pathobiont taxa (Enterococcaceae and Enterobacteriaceae) (Fig. 1a, Extended Data Fig. 1 and Supplementary Tables 2 and 3). To confirm that the observed microbiota differences were not due to the difference in median age between ICU and healthy cohorts, we also obtained publicly available datasets of 16s rRNA gene amplicon sequencing of rectal swabs from healthy volunteers with similar median age (62 years, range 42–80), sex and ethnicity distribution as our ICU patient cohort^22^. Again, the ICU microbiota displayed significantly different β-diversity, reduced α-diversity, as well as differential abundance and increased relative abundance of Enterobacteriaceae (Supplementary Fig. 1). Permutational multivariate analysis of variance (PERMANOVA) analysis identified that only biological sex and duration of antibiotic treatment before sampling were significantly associated with microbiota composition in the first week of critical illness, whereas age, ethnicity, burden of comorbidities (Charlson index), illness severity (sequential organ failure assessment (SOFA) score), duration of hospitalization before microbiota sampling and admission diagnosis (both subacute illnesses (sepsis) and hyperacute illnesses such as trauma, neurological injury and cardiac arrest) were not associated with microbiota composition (Extended Data Table 1 and Supplementary Fig. 2). All critically ill patients in this study were treated with enteral nutrition, none received stress-dose glucocorticoid therapy and one patient was treated with proton-pump inhibitor therapy during admission. Collectively, these data demonstrate that dysbiosis of the fecal bacterial microbiota is established at the time of ICU admission and exhibits dynamic and progressive changes during the acute phase of critical illness.

**Extended Data Fig. 2:**
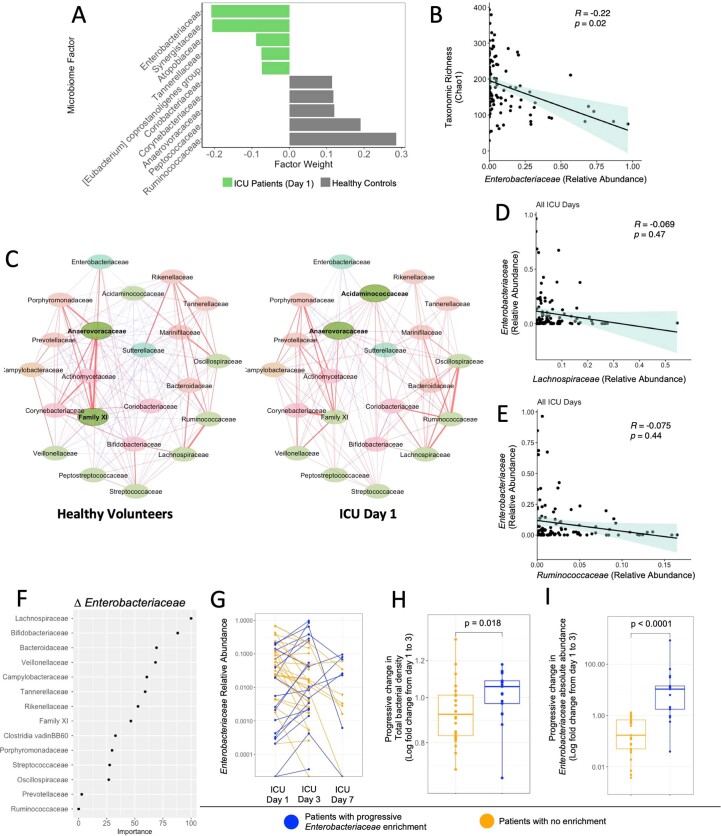
Inter-bacterial dynamics in the fecal microbiota of ICU patients. **(a)** Multi-Omics Factor Analysis (MOFA) was used to calculate the explained variance of microbiota composition between healthy volunteers and ICU patients on day 1 of admission, showing top 10 taxonomic factors (families) and their relative contributions to explained variance (factor weight). **(b)** Spearman correlation between the relative abundance of *Enterobacteriaceae* and taxonomic richness (Chao1) in ICU patients across all sampling timepoints. Dots show individual patient samples, regression line and 95% confidence interval (shaded area) shown. **(c)** Spearman correlation network of the most abundant bacterial families in healthy volunteers and ICU patients on day 1 of admission (upper quartile, 25%). **(d, e)** Spearman correlation between *Enterobacteriaceae* relative abundance and the relative abundance of *Lachnospiraceae* (D) and *Ruminococcaceae* (E) in ICU patients across all sampling timepoints. Dots show patient samples, regression line and 95% confidence interval (shaded area) shown. **(f)** Penalized ridge regression of the 15 most abundant bacterial families in ICU patients and their importance towards the progressive change in *Enterobacteriaceae* relative abundance from day 1 to 3 of admission (∆*Enterobacteriaceae*). **(g)** Relative abundance of *Enterobacteriaceae* on days 1, 3, and 7 of ICU admission in patients with (N = 18) or without (N = 26) progressive enrichment of *Enterobacteriaceae* in their fecal microbiota. **(h)** Progressive change in total bacterial density, and **(i)** *Enterobacteriaceae* absolute abundance in rectal swab samples from day 1 to 3 of admission (shown as log fold change) in patients with (N = 17, one patient had insufficient remaining sample for qPCR) or without (N = 26) progressive enrichment of *Enterobacteriaceae* in their fecal microbiota. Dots are individual patients, central line is median, box shows IQR, whiskers show range, statistical analysis by 2-sided Mann–Whitney test. P values as shown.

Using multi-omics factor analysis (MOFA)^23^ we found that variance in microbiome composition between critically ill patients and healthy volunteers was overwhelmingly explained by members of the Enterobacteriaceae family, both on admission as well as across all time points during the first week in ICU (Fig. 1e and Extended Data Fig. 2a). Proteobacteria, and in particular Enterobacteriaceae, expansion has been consistently observed in previous studies of hospitalized and critically ill patients^11–14,20,24^. Median Enterobacteriaceae relative abundance was ~tenfold higher in rectal swab samples of critically ill patients compared to healthy controls, with individual patient variability that was dynamic over the first week of ICU admission (Fig. 1f). Enterobacteriaceae abundance was inversely correlated with total microbiota richness and diversity (Fig. 1g and Extended Data Fig. 2b) and penalized ridge regression analysis revealed that Enterobacteriaceae was the most important family associated with the change in microbiota diversity over time in ICU patients (Fig. 1h). Increased Enterobacteriaceae relative abundance coincided with early reduction of anaerobic fermenters including Ruminococcaceae and Lachnospiraceae (Extended Data Fig. 1b,c). Community network visualization and Spearman correlation analyses between bacterial families did not reveal significant pairwise correlations between Enterobacteriaceae and anaerobic fermenters such as Ruminococcaceae, Lachnospiraceae and Bifidobacteriaceae at individual points in time (Extended Data Fig. 2c–e and Supplementary Tables 4–7). In contrast, longitudinal analysis of the change of Enterobacteriaceae relative abundance between admission (day 1) and day 3 of ICU using penalized ridge regression identified Lachnospiraceae and Bifidobacteriaceae as the most important families associated with Enterobacteriaceae dynamics (Extended Data Fig. 2f), which aligns with their known role in colonization resistance against Enterobacteriaceae expansion in the gut^25^.

Further interrogation of the temporal changes of Enterobacteriaceae over the first week of ICU admission demonstrated that 41% of patients with serial sampling had greater than doubling of Enterobacteriaceae relative abundance between consecutive sampling time points, hereafter referred to as progressive Enterobacteriaceae enrichment (14 of 18 between days 1 and 3 and 4 of 18 between days 3 and 7; Extended Data Fig. 2g and Supplementary Table 1). Notably, both univariable and multivariable regression analysis found that the development of progressive Enterobacteriaceae enrichment was independent of age, sex, comorbidities, admission diagnosis, antibiotic treatment (spectrum or duration before microbiota sampling), duration of hospitalization before microbiota sampling or illness severity (Extended Data Table 2). Progressive Enterobacteriaceae enrichment was not associated with expansion of other pathobionts, but was instead linked to a reduction in overall bacterial community stability (Fig. 1i–k). Furthermore, quantification of total fecal bacterial density by qPCR as well as total Enterobacteriaceae abundance (quantified by total bacterial density multiplied by relative abundance of Enterobacteriaceae, as previously reported^20,26^) revealed that patients with progressive Enterobacteriaceae enrichment had both progressive increase in total bacterial density and total Enterobacteriaceae quantity, indicating that enrichment was mediated by Enterobacteriaceae expansion rather than contraction of other taxa (Extended Data Fig. 2h,i). Together, these findings reveal dynamic and progressive microbiota injury in acute critical illness dominated by Enterobacteriaceae enrichment.

**Extended Data Fig. 3:**
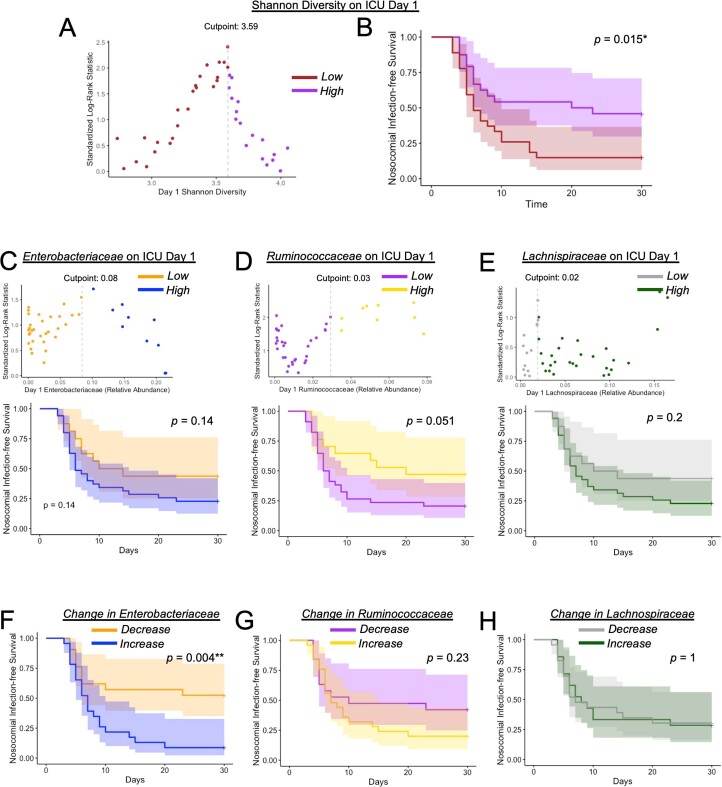
Microbiota dynamics and nosocomial infection-free survival in critical illness. **(a)** Maximally selected rank statistics identified that a Shannon diversity index value of 3.59 on day 1 of admission yielded the greatest separation of patients based on nosocomial infection-free survival. Patients with Shannon index above this cutoff (>3.59) were grouped as ‘high’ Shannon index, and those below cutoff (<3.59) were grouped as ‘low’ Shannon index. **(b)** Kaplan–Meier curve of nosocomial infection-free survival between patients with high Shannon diversity or low Shannon diversity as determined in (A). **(c–e)** Maximally selected rank statistics identified relative abundance values on day 1 of ICU admission of (C, top) *Enterobacteriaceae*, (D, top) *Ruminococcaceae*, and (E, top) *Lachnospiraceae* that yielded the greatest separation of patients based on nosocomial infection-free survival. Patients with relative abundance values above the cutoff were grouped as ‘high’, and those below cutoff were grouped as ‘low’ for each respective bacterial family. (Bottom panels) Kaplan–Meier curves of nosocomial infection-free survival between patients with high versus low relative abundance of (C, bottom) *Enterobacteriaceae*, (D, bottom) *Ruminococcaceae*, and (E, bottom) *Lachnospiraceae*. **(f–h)** Kaplan–Meier curves of nosocomial infection-free survival in patients stratified by the change in relative abundance between day 1 and 3 of ICU admission (increase vs decrease) of (F) *Enterobacteriaceae*, (G) *Ruminococcaceae*, and (H) *Lachnospiraceae*. Statistical analysis was performed using log-rank test, p values as shown.

Microbiota dysbiosis has been linked to adverse outcomes including nosocomial infections in hospitalized and critically ill patients^11,14,16,20,24,27^. Consistent with this, we found that patients with low microbiota Shannon diversity on admission (<3.59, cutoff determined by maximally selected rank statistics) had a significantly increased risk of nosocomial infection or death compared to patients with a high Shannon diversity (>3.59) on admission (Extended Data Fig. 3a,b). To explore whether this relationship between microbiota dysbiosis and nosocomial infection-free survival was associated with particular taxa (either quantity or temporal dynamics), we focused on bacterial families that were differentially abundant in ICU patients compared to healthy controls (Enterobacteriaceae, Ruminococcaceae and Lachnospiraceae; Extended Data Fig. 1). The relative abundance of these families at admission was not associated with nosocomial infection-free survival (Extended Data Fig. 3c–e). In contrast, patients who experienced any increase in Enterobacteriaceae relative abundance between time points were at significantly higher risk of infection or death compared to patients with decreased Enterobacteriaceae, whereas no association was observed for Ruminococcaceae or Lachnospiraceae dynamics (Extended Data Fig. 3f–h). Furthermore, patients with doubling or more of Enterobacteriaceae relative abundance between time points (which we define as progressive Enterobacteriaceae enrichment) were found to have significantly increased risk of the composite of nosocomial infection or death, as well as higher odds of nosocomial infection (OR 6.8, 95% CI 1.7–25.3) compared to patients without progressive Enterobacteriaceae enrichment (Fig. 1l,m). Members of the Enterobacteriaceae family are common causative pathogens in nosocomial infections and previous studies have suggested a direct link between gut overgrowth and infection via translocation and dissemination^11,15,17,24^. Clinical microbiology data identified Enterobacteriaceae organisms in 27% of nosocomial infections in this cohort of critically ill patients (Table 2); however, no significant association was found between progressive Enterobacteriaceae enrichment in the fecal microbiota and the odds of infection caused by Enterobacteriaceae pathogens (OR 0.97, 95% CI 0.2–4.7), although this analysis is likely underpowered due to the relatively small number of Enterobacteriaceae infections in this study (Fig. 1m). Instead, pathogens identified in nosocomial infections were diverse and not different between those with fecal Enterobacteriaceae enrichment and those without enrichment (Fig. 1n and Table 2). Therefore, microbiota dysbiosis and progressive Enterobacteriaceae enrichment are associated with an increased risk of nosocomial infections caused by a spectrum of bacterial and fungal pathogens, suggestive of a state of globally impaired host defense.

**Table 2:** Nosocomial infections in study participants

Patient	Progressive fecal Enterobacteriaceae enrichment	Infection diagnosis	Clinical microbiology diagnosis
44	Yes	VAP	*Staphylococcus aureus*, *Haemophilus influenzae*
20	Yes	VAP	*Escherichia coli*
42	Yes	VAP	*Pseudomonas aeruginosa*, *Haemophilus influenzae*
13	Yes	VAP and UTI	No pathogen identified (tracheal aspirate) *Enterococcus faecalis* (urine and secondary BSI)
27	Yes	VAP	Mixed bacterial growth not otherwise specified
45	Yes	VAP	Mixed bacterial growth not otherwise specified
46	Yes	VAP	Mixed bacterial growth not otherwise specified
51	Yes	VAP	No pathogen identified^a^
28	Yes	HAP	No pathogen identified
14	Yes	UTI	*Escherichia coli*
17	Yes	UTI	*Escherichia coli*
4	Yes	UTI	Mixed bacterial growth not otherwise specified
9	Yes	Peritonitis	*Escherichia coli*, *Bacteroides fragilis*
38	Yes	BSI	Coagulase-negative *Staphylococcus*
43	Yes	Diarrhea/colitis	*Clostridioides difficile*
22	No	VAP	*Pseudomonas aeruginosa*, *Klebsiella pneumonia*
40	No	VAP	*Staphylococcus aureus*
10	No	VAP	No pathogen identified
41	No	VAP	No pathogen identified
32	No	HAP	*Klebsiella pneumoniae*
21	No	HAP + UTI	*Escherichia coli*
18	No	HAP	No pathogen identified^b^
1	No	HAP	No pathogen identified
2	No	HAP	No pathogen identified
12	No	BSI	Coagulase-negative *Staphylococcus*
48	No	BSI	*Candida albicans*
11	NA	VAP	*Klebsiella oxytoca*
29	NA	VAP	Mixed bacterial growth not otherwise specified

BSI, bloodstream infection; VAP, ventilator-associated pneumonia; UTI, urinary tract infection, HAP, hospital-acquired pneumonia (non-ventilated).

^a^Growth of *C.* *paropsilosis* in endotracheal aspirate but not deemed causative pathogen.

^b^Growth of *C.* *tropicalis* in endotracheal aspirate but not deemed causative pathogen.

### Dysbiosis of a microbiota–immune metasystem in critical illness

**Fig. 2:**
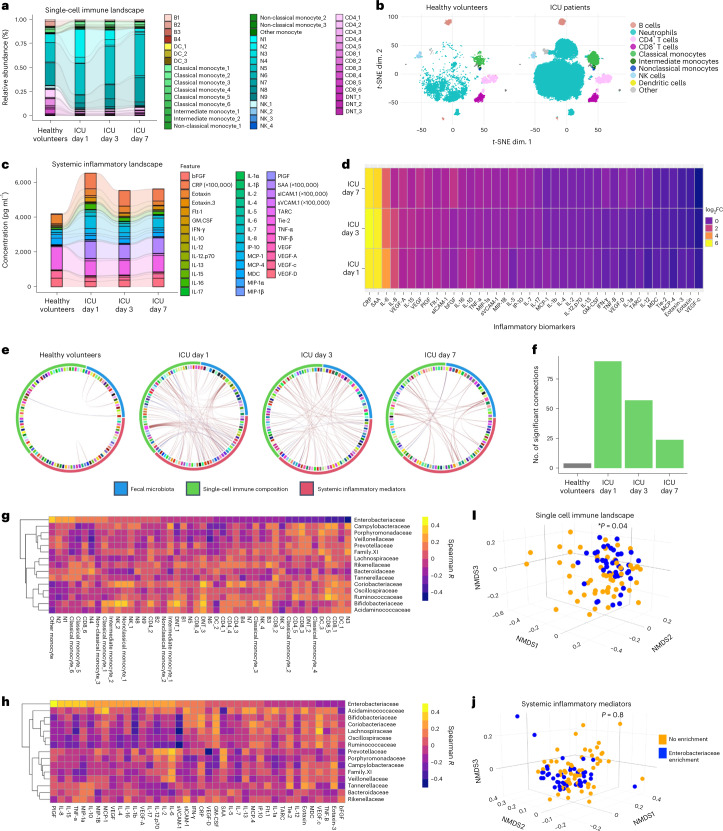
Dynamic microbiota–immune metasystem dysbiosis in critical illness. **a**–**d**, The cellular immune landscape of blood (**a**,**b**) and plasma inflammatory mediators (**c**,**d**) were quantified by mass cytometry and multiplexed electrochemiluminescence assays, respectively, in blood samples from critically ill patients (*n* = 51) sampled on day 1 of admission (*n* = 49) and again from survivors who remined in ICU on day 3 (*n* = 43) and day 7 (*n* = 15), compared to healthy volunteer controls (*n* = 12). The abundance of all immune cell populations (shown as %CD45^+^) identified by FlowSOM clustering of single-cell mass cytometry data (Methods) (**a**) and *t*-SNE dimensionality reduction of the single-cell immune landscape between healthy volunteers and ICU patients (**b**). Concentrations (pg ml^−1^) of inflammatory mediators in the plasma (**c**) and log_2_ fold change (FC) (**d**) in concentrations of each mediator in ICU patients on days 1, 3 and 7 compared to healthy volunteers. CRP, C-reactive protein; TNF, tumor necrosis factor; IFN, interferon; SAA, serum amyloid A. **e**,**f**, Chord diagrams depicting the significant Spearman correlations (false discovery rate (FDR)-adjusted *P* < 0.1) between microbiota composition, immune cell landscape and systemic inflammatory mediators in healthy volunteers and ICU patients at each time point (**e**) and quantification of the number of significant Spearman’s correlations (FDR-adjusted *P* < 0.1) between metasystem compartments (**f**). **g**,**h**, Heat map of individual Spearman’s correlation coefficients between the 15 most abundant microbiota families (relative abundance) and immune cell clusters in blood (**g**) and plasma inflammatory mediators (**h**) across the first week of ICU admission. **i**,**j**, NMDS ordination of the single-cell immune landscape (**i**) and systemic inflammatory mediators (**j**) across the first 7 d of ICU admission in patients with (*n* = 18 patients) and without (*n* = 26 patients) progressive fecal Enterobacteriaceae enrichment. Statistical comparisons were performed using PERMANOVA (Supplementary Tables 15 and 16 show full model results) accounting for repeated measures, each point represents an individual patient-time point; *P* values as shown. *t*-SNE, *t*-distributed stochastic neighbor embedding.

**Extended Data Fig. 4:**
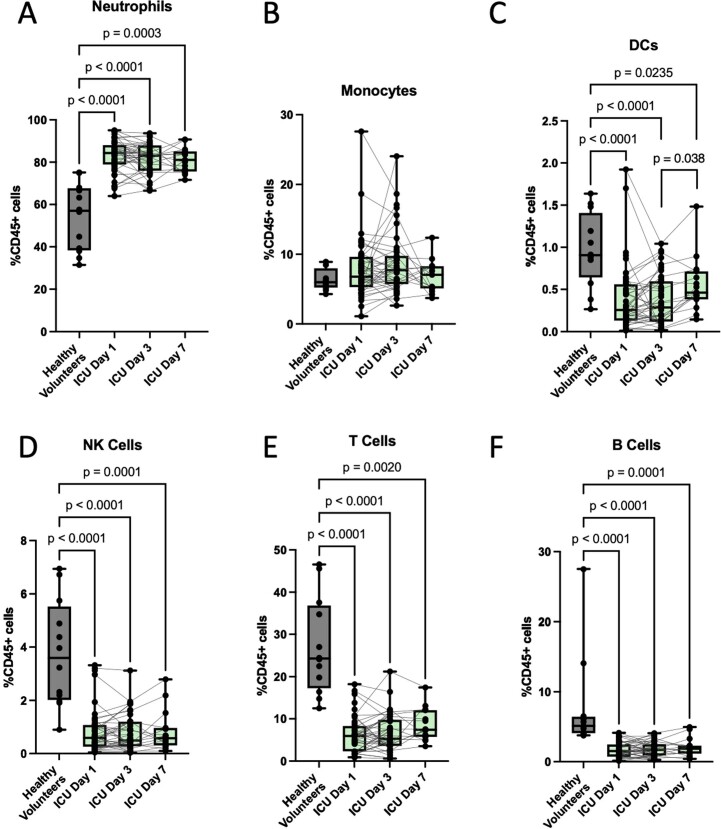
Immune cell dynamics in critically ill patients. Mass cytometry of whole blood was used to quantify the abundance of major immune cell populations in critically ill patients (N = 51) sampled on day 1 of admission (N = 49), and again from survivors who remined in ICU on day 3 (N = 43), and day 7 (N = 15), compared to healthy volunteer controls (N = 12). Data are shown as %CD45^+^ cells in blood for (**a**) neutrophil, (**b**) monocytes, (**c**) dendritic cells, (**d**) NK cells, (**e**) T lymphocytes, and (**f**) B lymphocytes. Dots are individual patients, central line is median, box shows IQR, whiskers show range. Statistical comparisons between healthy volunteers and ICU patients at each time point were performed using a Kruskal–Wallis test, while pairwise comparisons of repeated measures across ICU patient-days were performed using mixed linear regression model to account for repeated measures and variable drop-out, with post hoc Tukey’s tests. P values as shown.

**Extended Data Fig. 5:**
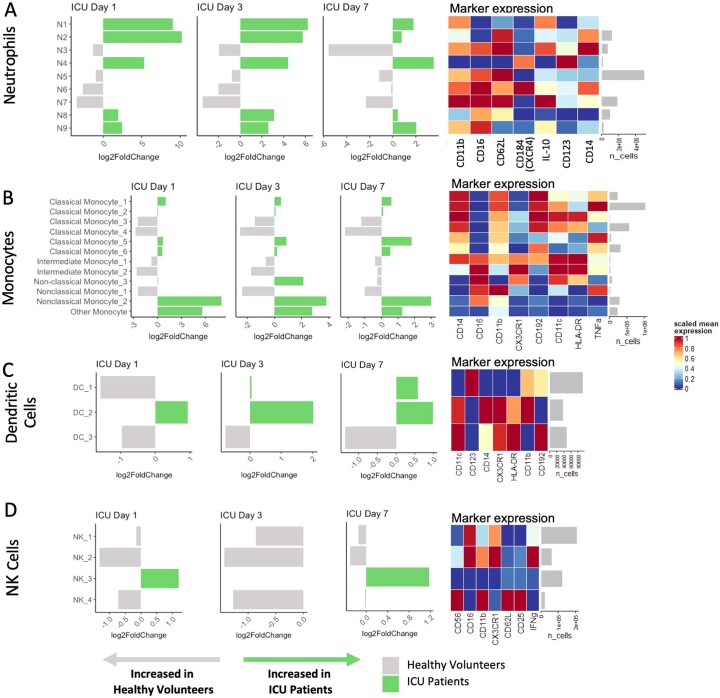
Innate immune landscape during critical illness. FlowSOM clustering of single-cell mass cytometry data was used to identify unique clusters of (**a**) neutrophil, (**b**) monocytes, (**c**) dendritic cell, and (**d**) NK cells in blood from critically ill patients (N = 51) sampled on day 1 of admission (N = 49), and again from survivors who remined in ICU on day 3 (N = 43), and day 7 (N = 15), compared to healthy volunteer controls (N = 12). Graphs shown log_2_ fold difference of cluster abundance between healthy controls and ICU patients on ICU day 1, day 3, and day 7. Heatmaps show mean expression level of key markers for each cell type measured by mass cytometry (scaled by column/marker), as well as total quantity (mean) of each cell cluster (cells/mL of blood).

**Extended Data Fig. 6:**
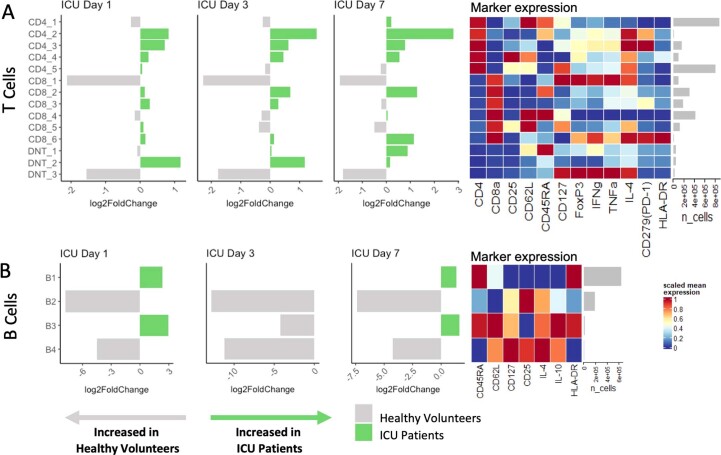
Adaptive immune cell landscape during critical illness. FlowSOM clustering of single-cell mass cytometry data was used to identify unique clusters of (**a**) T cells, and (**b**) B cells in blood from critically ill patients (N = 51) sampled on day 1 of admission (N = 49), and again from survivors who remined in ICU on day 3 (N = 43), and day 7 (N = 15), compared to healthy volunteer controls (N = 12). Graphs shown log_2_ fold difference of cluster abundance between healthy controls and ICU patients on ICU day 1, day 3, and day 7. Heatmaps show mean expression level of key markers for each cell type measured by mass cytometry (scaled by column/marker), as well as total quantity (mean) of each cell cluster (cells/mL of blood).

We next performed a systems-level analysis of the cellular immune and inflammatory landscapes in the bloodstream of each patient to test the hypothesis that microbiota injury in critical illness is coupled with impaired systemic immunity. High-dimensional single-cell analysis of the circulating immune landscape using mass cytometry revealed profound shifts in innate and adaptive immunity in critically ill patients that were dynamic over the first week of admission (Fig. 2a,b and Extended Data Fig. 4). Consistent with previous reports^3,4,28^, the cellular immune response in acute critical illness was dominated by an early and sustained elevation of neutrophils, together with depletion of T and B lymphocytes as well as natural killer (NK) cells (Extended Data Fig. 4). Clustering of single-cell data using FlowSOM revealed that neutrophil expansion in critically ill patients was attributed largely to immature neutrophils (CD16^lo/int^CD11b^lo/int^, clusters N1, N2 and N8) including a population resembling recently characterized dysfunctional CD123^+^ neutrophils (cluster N4)^29^, with reduction of mature (CD16^hi^CD11b^hi^, clusters N3, N5 and N7) and aged (CXCR4^+^CD62L^lo^, cluster N6) neutrophil populations (Fig. 2a, Extended Data Fig. 5a and Supplementary Table 8). Additional multi-lineage innate immune dysregulation was observed including monocyte dysregulation (early and sustained depletion of HLA-DR-expressing classical and intermediate monocyte clusters CM3, CM4, IM1 and IM2, as well as expansion of non-classical monocyte clusters NC2 and NC3), loss of HLA-DR^+^ dendritic cells (cluster DC3) and decreased activated interferon-γ^+^ NK cells (cluster NK2) (Extended Data Fig. 5b–d and Supplementary Table 8). Within the adaptive immune compartment, global T and B cell lymphopenia predominated in critically ill patients, with the remaining T cell pool enriched with PD-1^+^ CD4^+^ and CD8^+^ T cell clusters (CD4-2, CD4-3 and CD8-6) and regulatory T (T_reg_) cell (CD4^+^CD25^+^FoxP3^+^, CD4-4) clusters (Extended Data Fig. 6a,b and Supplementary Table 8). Quantification of circulating inflammatory mediators revealed acute and dynamic upregulation of pro-inflammatory (interleukin (IL)-6, tumor necrosis factor-α, IL-8, C-reactive protein and serum amyloid A) and anti-inflammatory (IL-10 and IL-4) responses (Fig. 2c,d and Supplementary Figs. 3 and 4) characteristic of a cytokine storm syndrome^30^. Collectively, these data reveal dynamic cellular immune and inflammatory responses in critically ill patients characterized by early innate immune dysregulation and systemic inflammation, followed by progressive innate and adaptive immune dysfunction.

Given the overlapping temporal dynamics of microbiota injury and systemic immune dysregulation during acute critical illness, we sought to determine whether microbiota and immune dynamics demonstrated metasystem-level connectivity. Using Chord diagram analysis and visualization of connectivity between microbial taxa and immune components, a higher number of significant interactions was observed in ICU patients at admission compared to the connectivity observed in healthy volunteers (Fig. 2e,f). Augmented microbiota–immune connectivity was sustained through the early phase of critical illness, remaining elevated on days 3 and 7 of admission (Fig. 2e,f). To identify whether this surge in microbiota–immune connectivity was linked to specific taxonomic changes in the microbiota, we quantified Spearman correlation coefficients between each of the 15 most abundant bacterial families and individual immune cell subsets (Fig. 2g and Supplementary Tables 9–11) and inflammatory mediators (Fig. 2h and Supplementary Table 12–14). Hierarchical analysis (indicated by the dendrogram) revealed that the associations between Enterobacteriaceae and both cellular and inflammatory mediators were unique compared to all other microbial families (Fig. 2g,h). Strong correlations were found between Enterobacteriaceae relative abundance and innate immune responses, with increased Enterobacteriaceae correlating with higher levels of immature neutrophils (clusters N1, N2 and N4) and classical monocytes (clusters CM5 and CM6) and reduced mature neutrophils (cluster N3) (Fig. 2g). Furthermore, increased Enterobacteriaceae positively correlated with prototypical systemic inflammatory mediators (IL-8, IL-15, tumor necrosis factor-α, MIP-1α and IL-10), whereas no correlations were found with acute phase reactants C-reactive protein and serum amyloid A (Fig. 2g and Supplementary Fig. 5a–d). Temporal analysis over the first week of critical illness revealed changes in the magnitude of correlations between Enterobacteriaceae and inflammatory and innate immune landscapes (Supplementary Figs. 5e,f and 6 and Supplementary Tables 9–14).

**Extended Data Fig. 7:**
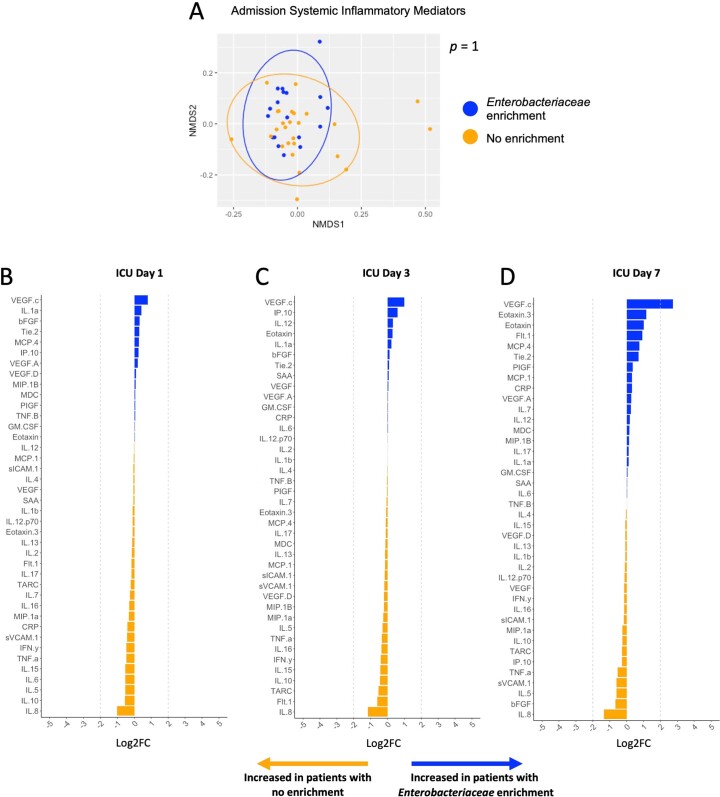
*Enterobacteriaceae* enrichment in the fecal microbiota and systemic inflammatory response during critical illness. **(a)** Non-metric multidimensional scaling (NMDS) ordination of the systemic inflammatory mediators at time of ICU admission in patients who subsequently developed (N = 18 patients) or did not develop (N = 26 patients) progressive fecal *Enterobacteriaceae* enrichment. Statistical comparisons were performed using permutational analysis of variance (PERMANOVA), each point represents an individual patient-time point, *p* value as shown. **(b–d)** Plasma levels of inflammatory mediators were compared (shown as log_2_ fold difference) between ICU patients with (N = 18) or without (N = 26) progressive enrichment of *Enterobacteriaceae* in their fecal microbiota on day 1 of admission, and again from survivors who remined in ICU on day 3, and day 7.

Consistent with these observations, dimensionality reduction of the single-cell immune landscape using non-metric multidimensional scaling (NMDS) revealed that patients with progressive Enterobacteriaceae enrichment (doubling or more of Enterobacteriaceae relative abundance during the first week in ICU) displayed cellular immune responses that differed significantly compared to those without progressive enrichment (Fig. 2i), even after controlling for patient covariables associated with immune cell composition including age, sex, admission diagnosis, ethnicity and illness severity (Supplementary Table 15). In contrast, no significant difference was observed in the circulating inflammatory mediator landscape between patients with and without progressive Enterobacteriaceae enrichment (Fig. 2j) as well as no association between the admission inflammatory mediator landscape and subsequent development of Enterobacteriaceae enrichment (Extended Data Fig. 7). Collectively, these data demonstrate that microbiota and cellular immune dynamics during acute critical illness function as an integrated metasystem and identify progressive Enterobacteriaceae enrichment as a possible driver of overall metasystem dysbiosis.

### Metasystem dysbiosis leads to a breakdown of innate immune defense

**Fig. 3:**
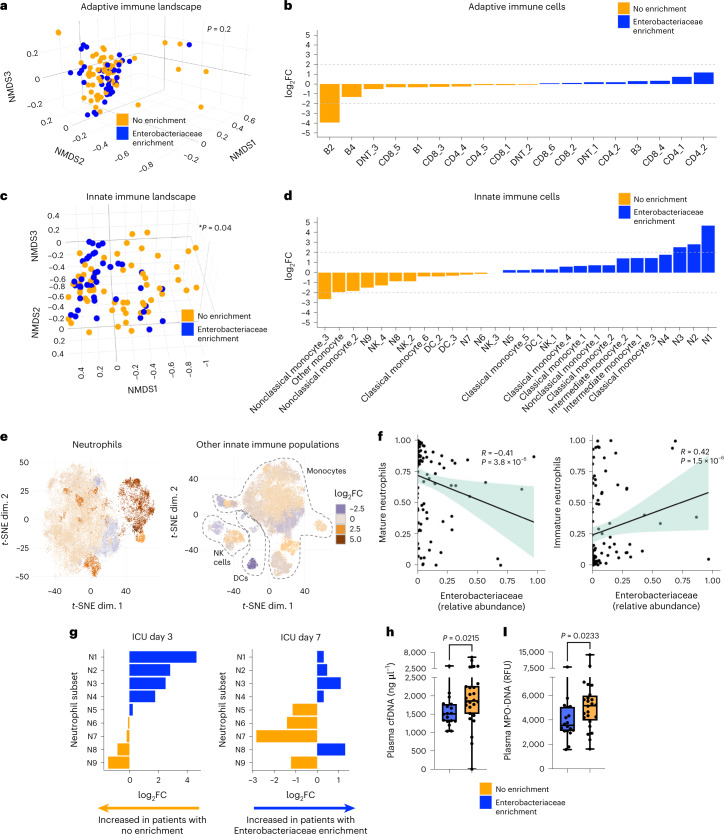
Enterobacteriaceae dysbiosis and impaired neutrophil host defense in critical illness. **a**–**d**, NMDS ordinations (**a**,**c**) and comparisons of abundance (**b**,**d**) of adaptive immune cell (T and B cells) populations and innate immune cell populations (all neutrophils, monocytes, dendritic cells and innate lymphoid cell populations) (**a**,**b**) identified by clustering of mass cytometry data in the blood of ICU patients with (*n* = 18) or without (*n* = 26) progressive enrichment of Enterobacteriaceae in their fecal microbiota. Dots show individual patient-time points across the first 7 d of ICU admission, with statistical analysis by PERMANOVA accounting for repeated measures (**a**,**c**). **e**, *t*-SNE plots of neutrophils (left) and all other innate immune cells (right; monocytes, dendritic cells and NK cell clusters as indicated), with heat map overlay showing the log_2_FC in abundance of each cell cluster between ICU patients with (*n* = 18) or without (*n* = 26) progressive enrichment of Enterobacteriaceae in their fecal microbiota. **f**, Correlation between fecal Enterobacteriaceae relative abundance and the quantity of mature (left) and immature (right) neutrophils (shown as proportion of total neutrophils) in ICU patients across the first week of admission analyzed using Spearman’s ranked correlation test. Dots show individual patient samples, regression (line) and 95% confidence intervals (shaded area) are shown. **g**, Comparison of neutrophil clusters in blood of ICU patients with (*n* = 18) or without (*n* = 26) Enterobacteriaceae enrichment (shown as log_2_ fold difference of cluster abundance between groups). To determine the independent contribution of Enterobacteriaceae enrichment status (**a**–**e**,**g**), analyses controlled for clinical covariables that were independently associated with immune cell composition (Supplementary Table 15). **i**,**j**, Quantification of plasma NET markers (**i**) cell-free DNA and (**j**) MPO–DNA complexes on ICU day 3 in patients with (*n* = 18) or without (*n* = 26) Enterobacteriaceae enrichment. Dots represent individual patients, central line shows the median, box shows the IQR and whiskers show the range; statistical comparison was performed using a two-sided Mann–Whitney *U*-test. *P* values are shown.

Next, we investigated whether Enterobacteriaceae-associated metasystem dysbiosis was characterized by defects in specific immune defense programs that may contribute to the elevated risk of bacterial and fungal nosocomial infections. Dimensionality reduction analysis revealed that the adaptive immune cell compartment in patients with progressive Enterobacteriaceae enrichment was not significantly different from those without (Fig. 3a). Aside from a single naive B cell population (cluster B2), there was little impact on lymphocyte responses in patients with progressive Enterobacteriaceae enrichment during the first week of critical illness (Fig. 3a,b). In stark contrast, the innate immune cell landscape was significantly different in patients with progressive Enterobacteriaceae enrichment in the fecal microbiota compared to those without enrichment (Fig. 3c,d). Analysis of individual innate immune cell clusters revealed that this difference was characterized primarily by large shifts in neutrophil clusters, with more limited impact on monocytes, dendritic cells and innate lymphocytes (Fig. 3d,e).

**Extended Data Fig. 8:**
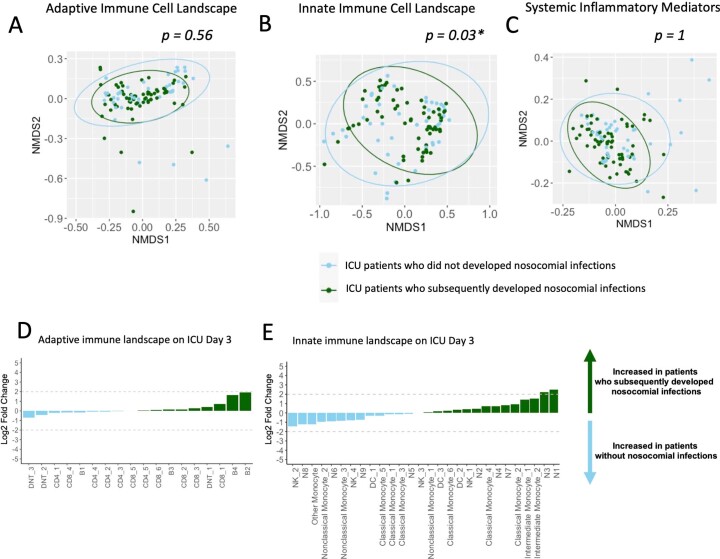
Cellular immune and inflammatory landscapes preceding nosocomial infections in critical illness patients. Non-metric multidimensional scaling (NMDS) ordination of the quantities across the first week of ICU admission of **(a)** adaptive immune cell (T and B cells) populations, **(b)** innate immune cell populations (all neutrophils, monocytes, dendritic cells, innate lymphoid cell populations) identified by unsupervised clustering of single cell mass cytometry analysis of blood, and **(c)** the systemic inflammatory mediators in patients who subsequently developed nosocomial infections (N = 28 patients) versus those who did not develop infections (N = 23 patients). Statistical comparisons were performed using permutational multivariate analysis of variance (PERMANOVA), each point represents an individual patient-time point, *p* values as shown. To determine the independent associations of nosocomial infection status, analyses controlled for clinical covariables that were independently associated with immune cell composition (Supplementary Table 15). **(d, e)** Differential abundance analysis of the **(d)** adaptive immune cell clusters and **(e)** innate immune cell clusters identified by unsupervised clustering of single cell mass cytometry analysis of blood from ICU on Day 3 of admission (prior to any nosocomial infections) in patients who subsequently developed nosocomial infections (N = 28 patients) and those who did not (N = 23 patients).

Previous studies using mouse models have reported an important role for the gut microbiota in directing neutrophil-mediated host defense via regulation of granulopoiesis, maturation and aging of circulating neutrophils^31–34^. Strong correlations were observed in ICU patients between Enterobacteriaceae relative abundance and increased immature neutrophils (CD16^lo/int^CD11b^lo/int^ clusters N1, N2, N4 and N8) and decreased mature neutrophils (CD16^hi^CD11b^hi^ clusters N3, N5, N7 and N9) (Fig. 3f). Despite similar total numbers of circulating neutrophils, ICU patients with progressive Enterobacteriaceae enrichment in their fecal microbiota had a notable shift in the landscape of neutrophils over time, including early and sustained increases in immature clusters (N1, N2 and N4) (Fig. 3g). Consistent with the temporal directionality between microbiota–immune metasystem dysbiosis and subsequent risk of nosocomial infections, we also compared the immune landscape between patients who developed nosocomial infections versus those who did not and again found that differences in the innate immune cell landscape, including expansion of immature neutrophils, preceded the development of infections (Extended Data Fig. 8). Recent studies have shown that immature neutrophil populations in humans display hypofunctional pathogen killing mechanisms, including impaired production of neutrophil extracellular traps (NETs)^35^. Patients with progressive Enterobacteriaceae enrichment (and associated immature neutrophil expansion) were found to have reduced quantities of circulating NET markers in their plasma (both cell-free DNA and myeloperoxidase (MPO)–DNA complexes) compared to those without enrichment (Fig. 3h,i). Overall, these findings reveal that increased susceptibility to nosocomial infections in the setting of progressive Enterobacteriaceae enrichment is coupled to dysregulated and hypofunctional neutrophil responses.

## Discussion

Here we show that the intestinal microbiota and systemic immune response of acute critical illness are functionally integrated as a dynamic metasystem. Dysbiosis of this metasystem in critical illness is associated with progressive Enterobacteriaceae expansion in the gut microbiota, dysregulated innate immunity and increased incidence of bacterial and fungal nosocomial infections.

Evidence from mouse models has shown that gut microbes regulate various mechanisms of systemic immunity and that germ-free and antibiotic-conditioned mice display impaired defense against a variety of bacterial, fungal and viral infections^33,34,36–39^. In critically ill humans, we observed that gut microbiota dysbiosis was coupled predominantly with innate immune dysregulation, with less impact on the landscape of adaptive immunity. In particular, the dominant response of neutrophils to microbiota alterations during the early stages of critical illness may reflect their rapid turnover and greater capacity for acute functional plasticity compared to the more protracted time course of adaptive immune responses. As our study focused on the first week of critical illness, it is possible that intestinal dysbiosis may drive adaptive immune dysregulation later in the course of illness, as previous studies have documented widespread lymphocyte apoptosis and functional exhaustion that persists in some patients and contributes to ongoing organ dysfunction and opportunistic nosocomial infections^3,6^. The impact of metasystem dysbiosis on long-term imprinting of both innate and adaptive immunity remains to be explored, but a previous retrospective study of hospitalized patients suggested that intestinal dysbiosis may be associated with a prolonged increased risk of infections and recurrent sepsis that lasts for months (or longer)^40^.

Gut microbes have been implicated in systemic neutrophil homeostasis through regulation of their maturation, aging, modulation of effector functions, as well as granulopoiesis in the bone marrow^31–34^. In mice, intestinal dysbiosis or germ-free status leads to suppressed myelopoiesis, impaired neutrophil maturation and defective antimicrobial effector functions^31–33,41^. Consistent with this, we observed a profound shift in neutrophil responses in patients with intestinal dysbiosis and progressive Enterobacteriaceae enrichment, marked by immature and hypofunctional neutrophil responses, including decreased NETs. This mechanism of microbiota–immune metasystem dysbiosis involving a core program of innate host defense is consistent with the elevated risk of systemic infections caused by diverse bacterial and fungal pathogens across multiple organs.

The ecological pressures driving microbiota dysbiosis in critical illness are likely multifactorial, including host intrinsic factors (age, comorbidities, physiological alterations of gut motility, mucosal blood flow, pH and others) as well as host-extrinsic factors that are inherent to critical illness (altered nutritional intake, medications (including antibiotics), invasive devices and ICU environment). Of note, we and others have found that abnormalities of community composition, diversity, as well as expansion of pathobionts such as Enterobacteriaceae are present from the time of ICU admission^10,11,20^. Notably, this was observed in patients with both subacute pre-ICU illnesses (such as sepsis) as well as more acute presentations (trauma, neurological injury and cardiac arrest) and was present despite our stringent exclusion criteria that limited pre-ICU hospitalization or antibiotic exposure. Following admission to ICU, we observed progressive and dynamic changes to the microbiota during the acute phase of critical illness that was characterized by expansion of Enterobacteriaceae and reduction in anaerobic fermenters, consistent with evidence implicating anaerobic fermenters in colonization resistance against Enterobacteriaceae^42^. Notably, among the multi-taxa changes observed during acute critical illness, only progressive Enterobacteriaceae enrichment (but not reduced anaerobic fermenters such as Ruminococcaceae or Lachnospiraceae) was associated with impaired host defense and risk of nosocomial infections, suggesting that Enterobacteriaceae exerts a unique influence on the microbiota–immune metasystem during critical illness. A recent study of ICU patients found that expansion of Enterobacteriaceae in the gut in the setting of anti-anaerobic antibiotic administration was also coupled with an increased risk of nosocomial infections^20^. These findings raise the possibility that interventions such as antibiotics may propagate a vicious cycle of microbiota–immune metasystem dysbiosis through suppression of gut anaerobe-mediated colonization resistance and expansion of Enterobacteriaceae. Of note, intestinal Enterobacteriaceae expansion has been observed in other hospitalized non-ICU patient populations who are at risk of nosocomial infections and therefore our findings may have implications beyond critical illness.

Last, our findings may have important therapeutic implications for prevention and treatment of infections in the ICU. Nosocomial infections remain a leading cause of adverse outcomes in critical illness including mortality, prolonged hospitalization and resource utilization^1,2,43^. The association between microbiota dysbiosis and nosocomial infections has led to clinical trials of microbiota-modifying therapies in critically ill patients, but studies have been hindered by a limited understanding of the mechanisms linking dysbiosis with infections, resulting in untargeted approaches such as probiotics and digestive tract decontamination that have yielded modest or negligible benefits^44,45,46^, as well as possible harm in this vulnerable patient population^47–49^. Our findings reveal that pathological microbiota alterations in critical illness may render the host more susceptible to infections via induction of immune dysfunction, suggesting that microbiota therapeutics in the ICU should be targeted at correcting the drivers of microbiota–immune metasystem dysbiosis. We have identified intestinal Enterobacteriaceae expansion as a marker of metasystem dysbiosis in critical illness and therefore precision editing of intestinal Enterobacteriaceae colonization^50^ may represent a strategy to reduce infections and adverse outcomes by fortifying systemic immune defenses.

This study has a number of limitations, including a single-center design, limited sample size and inherent heterogeneity of critically ill patients (including diverse comorbidities and treatments). While our prospective and longitudinal analysis enabled the identification of notable associations with temporal directionality between microbiota–immune metasystem dysbiosis and subsequent nosocomial infections, we cannot definitively show causality. In addition, our interrogation of systemic immunity was limited to the bloodstream and microbiota analysis was limited to the rectal compartment, due to safety and ethical considerations of obtaining invasive tissue biopsies or endoscopic sampling of the proximal GI tract in critically ill patients. Furthermore, 16s sequencing and mass cytometry analysis provide detailed, albeit incomplete resolution of microbiota dysbiosis and immune cell heterogeneity. Therefore, future studies using expanded immune analysis and deeper microbiota sequencing may uncover additional mechanisms of immune dysfunction involving neutrophils and other antimicrobial effector cells and microbial alterations of the gut or other sites (lungs, oropharynx, skin or even ICU environment) that contribute to metasystem dysbiosis and impaired host defense in critical illness.

## Methods

### Study design and participants

This study was approved by the conjoint health research ethics board of the University of Calgary and Alberta Health Services (REB18-1294). Written informed consent was obtained from all study participants or appropriate surrogate decision maker for patients who were unable to provide consent due to incapacitating illness. Enrollment occurred between 23 July 2019 and 20 July 2021, with substantial delays and disruptions in enrollment due to the COVID-19 pandemic between March 2020 and April 2021. Patients admitted to the medical, surgical, neurological and trauma ICUs at the Foothills Medical Center in Calgary were screened for the following inclusion criteria (adapted from elsewhere^44^): adult (>18 years of age) with an index admission to ICU, requiring mechanical ventilation, who was expected to require continuous mechanical ventilation for >72 h as judged by the treating ICU specialist. Patients were excluded if they had a pre-existing immunocompromised state (systemic immunomodulatory therapy, chemotherapy, HIV infection or other congenital or acquired immunodeficiency), had been hospitalized >48 h before ICU admission in the previous 3 months, had received systemic antimicrobial therapy in the previous 3 months, had inflammatory bowel disease or active GI malignancy, previous surgery leaving a discontinuous GI tract, pregnancy, goals of care that excluded life-support interventions or moribund patients not expected to survive >72 h. At the onset of the COVID-19 pandemic, the study team added SARS-CoV-2 infection as an exclusion criterion and therefore no patients with COVID-19 were included in this study.

Rectal swabs and blood samples were collected from prospectively enrolled patients on the day of ICU admission (*n* = 51) and again from survivors who remained in the ICU on day 3 (*n* = 45) and day 7 (*n* = 18) following admission. For reference comparisons, rectal swabs and blood samples were also collected from healthy volunteers (*n* = 18) for use as controls. As a discovery-based study of microbiota–immune interactions, there were no previously published effect size estimates to facilitate an a priori sample size calculation, therefore we enrolled a cohort size that was comparable to other recently published multi-omic studies in critically ill patients^51–55^, as well as human microbiota–immune omics studies^56–58^. The number of patient samples included in each analysis is indicated in figure legends and varies slightly due to rare instances where the quality or quantity of individual samples were unsuitable for certain assays and could not be re-collected. Patient demographic and clinical data were collected at the time of admission and are displayed in Table 1. Clinical outcomes of nosocomial infection and death were recorded up to 30 d following ICU admission. Nosocomial infections were identified as newly diagnosed infections occurring at least 48 h after admission, diagnosed by the treating specialist physician, resulting in administration of new antimicrobial treatments and were independently confirmed by a physician member of the study team based on the following definitions: diagnosis of VAP and HAP required the presence of new or progressive radiographic infiltrate on chest radiograph plus two of fever, purulent sputum, white blood cell count >10 × 10^6^ l^−1^ or <3.0 × 10^6^ l^−1^, as previously described^44^ and all cases of VAP met the Center for Disease Control and Prevention National Healthcare Safety Network (CDC NHSN) case definition of probable or definite VAP^59^. Nosocomial BSIs met the CDC NHSN definition of BSI/central-line-associated BSI, UTIs were identified based on Infectious Disease Society of America clinical practice guideline criteria for the diagnosis of catheter-associated urinary tract infections and *Clostridium* *difficile* infection was based on the presence of new diarrhea and positive stool testing for *C.* *difficile* toxin^60,61^. The sites of nosocomial infection and clinical microbiology data are presented in Table 2. Of note, all nosocomial infections reported in this study were assumed to be acquired after ICU admission/enrollment (see exclusion criteria above) and are therefore separate from the admission diagnoses listed in Table 1 (in particular, admission diagnoses of sepsis in Table 1 were not caused by nosocomial infections, as all were due to community-acquired infections as shown in Supplementary Table 1).

### 16S rRNA gene amplification and sequencing

All experimental analyses were performed at the University of Calgary. Rectal swabs were collected and stored in sterile tubes at −80 °C. DNA was isolated using the DNeasy PowerSoil (QIAGEN) following the manufacturer’s protocol. Negative control swabs were processed identically and run through the study protocol as controls. PCR amplification of the 16S V4 region was performed using previously described dual indexed primers with sample barcodes and sequencing adaptors and PCR conditions^62^. PCR products were cleaned and size selected using Nucleomag beads (Macherey Nagel) following manufacturer’s instructions. Individual sample libraries were normalized using a SequalPrep Normalization Plate (Invitrogen), after which samples were pooled to create the final library. Quality control of the pooled next-generation sequencing library was performed using an Agilent Technologies 2200 TapeStation and Qubit dsDNA analyzer. The pooled 16S V4 amplicon library was sequenced using an Illumina MiSeq platform to produce 2 × 250-bp paired-end reads.

### 16S amplicon sequence data processing and analysis

De-multiplexed Illumina MiSeq paired-end reads (FASTQ) were processed in R v.4.1.2 following the DADA2 pipeline v.1.14. Forward and reverse reads were truncated to 230 bp and 210 bp, respectively or to the first base with a quality score Q < 2. Reads containing any ambiguous (N) nucleotides or reads containing more than two errors were removed. Samples with fewer than 1,000 reads following trimming and filtering steps were discarded from further analysis. Taxonomy of unique amplicon sequence variants (ASVs) was assigned in DADA2 by the RDP Classifier using the SILVA v.138.1 database. ASVs and sample data were combined using the Phyloseq package v.1.38.0 for further downstream analysis. Potential contaminants were identified and removed by the Decontam package v.1.14.0 based on the distributed frequency of ASVs and the DNA concentration of individual samples^63^. ASVs taxonomically assigned to Cyanobacteria, mitochondria or chloroplast were removed. Publicly available datasets of healthy volunteers (*n* = 95, without colonic polyps) from Dadkhah et al.^22^ were processed on raw FastQ files as described above.

Microbiome α-diversity metrics were calculated using the Microbiome package v.1.16.0. Community dissimilarity (β-diversity) was calculated on the Bray–Curtis dissimilarity measure by PERMANOVA using the adonis function in Vegan v.2.6 and three-dimensional visualization was performed by plotting the first three dimensions of the Bray–Curtis dissimilarity ordination using the plotly package v.4.10.0. Spearman correlation matrices were performed between the 15 most abundant bacterial families present in at least 10% of samples using the rcorr function in the Hmisc package v.4.7 and visualized using the ggplot2 package v.3.3.6 in R. Taxonomy plots showing relative abundances were plotted using ggplot2 package microbiomeutilities v.1.00.16 and the Phyloseq packages v.1.38.0 in R. Penalized ridge regression analysis (glmnet R package v.4.1-4) was used to determine the importance of the 15 most abundant bacterial families toward specified microbiome outcomes (change in Shannon diversity or change in Enterobacteriaceae relative abundance between ICU days 1 and 3) using a threefold cross validation repeated ten times through the R package caret v.6.0-93. Results were visualized using the vip package v.0.3.2 in R. Community stability was determined using the codyn R package on data from days 1 and 3 of ICU admission. Differential abundance analysis was performed using ANCOM-II on relative abundances, with patients fit as a random effect to account for repeat measures using the microbiomeMarker R package v.1.0.2. Network analysis was performed on bacterial families using the NetCoMi R package^64^ v.1.1.0 and shows Spearman correlations >0.2 of the most abundant (upper quartile 25%) bacterial families in all samples. Data are reported as per the STORMS (Strengthening the Organization and Reporting of Microbiome Studies) guidelines (Supplementary Table 18).

### Quantitative analysis of fecal bacterial density

Bacterial density measurements were performed by qPCR using a universal 16s rRNA gene primer set (forward, 5′-TCCTACGGGAGGCAGCAGT-3′; reverse, 5′-GGACTACCAGGGTATCTAATCCTGTT-3′) as previously described^65^ and a standard curve was generated from *Escherichia* *coli* strain Xen14 DNA (PerkinElmer). The PCR reaction was performed using the PowerUp SYBR Green kit (Thermo Fisher) on a StepOnePlus Real-Time PCR System (Thermo Fisher). Cycle conditions were as follows: 50 °C 2 min, 95 °C 10 min, 95 °C for 15 s (40 cycles) and 60 °C for 1 min. Determination of Enterobacteriaceae abundance was performed as previously reported by Chanderraj et al.^20^ by multiplying the absolute bacterial density by Enterobacteriaceae relative abundance determined by 16s rRNA gene amplicon sequencing.

### Time-of-flight mass cytometry

Whole blood samples used for mass cytometry analysis were cryopreserved in PROT1 proteomic stabilizer (SmartTube) at a ratio of 1:1.4 and stored at −80 °C to enable batched analysis of patient samples as described^58^. Samples were thawed at room temperature and red blood cell lysis was performed using PROT1 RBC lysis buffer (SmartTube) and white blood cells were washed in cell staining medium (PBS with 1% BSA) followed by labeling with a custom metal-conjugated antibody panel (Supplementary Table 17). White blood cells were incubated with metal-conjugated surface antibodies, followed by fixation and permeabilized (BD Cytofix-Cytoperm), incubation with intracellular antibodies, then left overnight in a solution containing Cell-ID iridium intercalator (Fluidigm), 0.3% saponin and 1.6% paraformaldehyde in PBS. Cells were then mixed with EQ Four Element Calibration Beads (Fluidigm) and acquired on a Helios CyTOFII mass cytometer (DVS). Mass cytometry data were normalized using the internal Helios CyTOFII bead-based normalization software (DVS).

### Single-cell mass cytometry data processing and analysis

Normalized mass cytometry data files were further processed in R using the CytoSpill package v.0.1.0 to correct for any signal overlap between markers^66^. Next, corrected FCS files were imported into Cytobank (Cytobank) for manual gating on CD45^+^ single-cell events and major cell populations (Supplementary Table 8). Manually gated events were then exported as FCS files for further analysis in R using the CATALYST package v.1.16.0. Batch correction was performed using the RemoveBatchEffect function in the limma package v.3.48.3. Gated cell populations were clustered based on the expression of all available markers in CATALYST using the FlowSOM function. Extremely rare metaclusters (<0.5% of events in each population) or aberrant clusters (aberrant expression of all panel markers or less than three panel markers) were also removed. *t*-SNE dimensionality reduction was performed on 1,000 randomly selected events from each sample using a perplexity of 80 for 5,000 iterations. Figures were generated within the built-in functions of CATALYST in R. Visualization of dimensionality-reduced cellular immune landscapes between study participants was performed using NMDS of the relative abundance of immune cell populations using the Vegan metaMDS function in R. Figures showing log_2_FC of individual cell populations (between healthy volunteers and ICU patients or between ICU patients with or without Enterobacteriaceae enrichment) were calculated on absolute cell counts and controlled for clinical covariables that were significantly associated with immune cell composition (Supplementary Table 15), using the DESeq2 package v.1.34.0.

### Plasma inflammatory mediator quantification and analysis

Cryopreserved plasma samples were used for quantification of inflammatory cytokines, chemokines and biomarkers using the V-PLEX Human Biomarker 40-Plex kit (MesoScale Diagnostics). Cell-free DNA levels were quantified using the Quanti-iT PicoGreen kit (Invitrogen) according to the manufacturer’s instructions and quantification of MPO–DNA complexes in plasma was performed as previously described^55^. Differential abundance of 40-plex plasma inflammatory biomarkers was performed on the log_2_-transformed concentration values using the limma package v.3.48.3 in R, as previously described^67^. Dimensionality reduction and visualization of the differences in plasma inflammatory mediators between patients with and without microbiota Enterobacteriaceae enrichment was performed using NMDS of mediator concentrations using the Vegan package v.2.6 in R. Figures showing log_2_FC of individual mediators between ICU patients with or without Enterobacteriaceae enrichment were calculated on absolute concentration using the limma package v.3.48.3 and plotted with ggplot2 . No clinical covariables were significantly associated with inflammatory mediator landscape composition (Supplementary Table 16).

### Multi-omics integration and analysis

To perform an integrated multi-omics analysis of the fecal microbiota, cellular immune composition and inflammatory mediator landscape in blood, we employed an unsupervised factor analysis approach in R with MOFA v.1.4.0, as previously described^68^. Briefly, microbiota taxonomic data were aggregated to the family level and filtered to a cutoff of 25% prevalence, after which the count data was transformed by center log ratio (clr). Single-cell mass cytometry count data and inflammatory mediator concentrations were log transformed before dataset integration. The resulting MOFA factors were then compared between healthy volunteers and ICU patients to determine latent factors that explain variation between these populations. Modeling of the combined datasets in MOFA was performed using the default parameters with model fitting identifying the top ten MOFA factors that explained the largest amount of variation between samples. Next, MOFA factors showing contributions from all meta-systems datasets (fecal microbiota, single-cell immune composition and systemic inflammatory mediators) that explained at least 5% of variance were compared between healthy volunteers and ICU patients. Within each MOFA factor, individual features weights (such as individual microbial taxa within microbiota factors) were compared between healthy volunteers and ICU patients.

Connectivity between microbiota, cellular immune landscape and inflammatory mediators was determined using Chord diagram analysis. A Spearman correlation coefficient was calculated for pairings of all microbial taxa (10% prevalence cutoff, relative abundance and family level), immune cell subset counts and inflammatory mediatory concentrations (FDR-adjusted for multiple comparisons with *P* < 0.1) and significant values were visualized using the circlize package v.0.4.15 in R. Heat maps depicting the Spearman correlation coefficients between the 15 most abundant bacterial families (10% prevalence cutoff) and immune components (cell counts and inflammatory mediator concentrations) were generated using the rcorr function in the Hmisc package v.4.7 and visualized using the pheatmap package v.1.0.12 in R.

### Statistical analysis

Microbiota α-diversity metrics (Shannon index and Chao1), as well as relative abundance data of individual bacterial families were analyzed between healthy volunteers and ICU patient days using a Kruskal–Wallis test with a post hoc Tukey test. Analysis of paired measurements from ICU patients across days 1, 3 and 7 of admission was performed using a linear mixed-effects model to account for repeated measures and variable dropout across sampling time points using the lmerTest package v.3.1.3 in R (no additional clinical covariables were included in these models). A post hoc Tukey test was performed on the model for comparison between days in ICU using the emmeans package v.1.7.4 in R. Microbiota β-diversity was calculated on the Bray–Curtis dissimilarity by PERMANOVA using the adonis function in the Vegan package v.2.6, with pairwise ANOVA comparisons between healthy volunteers and ICU patient days using the EcolUtils package v.0.1 in R. To determine the associations between microbiota β-diversity and patients demographic and clinical covariables (categorical variables of biological sex, admission diagnosis and ethnicity; and continuous variables of age, comorbidities Charlson index, SOFA score, duration of hospitalization and antibiotic treatment before microbiota sampling), multivariable permutational ANOVA was performed in Vegan v.2.6. in R with permutations blocked by patients to account for repeated measures across sampling time points, with results shown in Extended Data Table 1.

Statistical analyses of NMDS of immune cell population abundances and inflammatory mediators between patients with and without progressive Enterobacteriaceae enrichment were performed using multivariable PERMANOVA in Vegan v.2.6. To account for repeated measures for multi-time-point comparisons, permutations were blocked by patients. To determine the associations between demographic and clinical covariables and control for their effects on immune cell composition and inflammatory mediator landscapes, both categorical variables (biological sex, admission diagnosis and ethnicity) and continuous variables (age, comorbidities Charlson index, SOFA score, duration of hospitalization and antibiotic treatment before microbiota sampling) were included in the models and are shown in Supplementary Tables 15 and 16.

To investigate associations between the outcome of progressive Enterobacteriaceae enrichment in ICU patients and demographic/clinical covariables known to influence microbiota and/or immune composition, we performed both univariable and multivariable analyses, with full model results shown in Extended Data Table 2. Univariable analyses were performed by Wilcoxon rank-sum test for continuous variables and Fisher’s exact test for categorical variables and multivariable analysis was performed using logistic regression to determine the association between demographic/clinical variables and the outcome of progressive Enterobacteriaceae enrichment.

The 30-d nosocomial infection-free survival data were visualized using a Kaplan–Meier curve and analyzed using a log-rank (Mantel–Cox) test. Maximally selected rank statistics were utilized to define cutoff values for microbiota variables (admission Shannon diversity, admission relative abundance or progressive change in relative abundance from day 1 to 3 of Enterobacteriaceae, Ruminococcaceae or Lachnospiraceae) to identify maximal separation of patients based on 30-d nosocomial infection-free survival using the surv_cutpoint function in the survminer R package v.0.4.9. To avoid bias from exclusion of zero values, relative abundance values of zero were replaced with a value of 0.00001 (half the lowest abundance value). Odds ratios were calculated and analyzed using a two-sided Fisher’s exact test.

All statistical analyses were performed in R or GraphPad Prism v.9.3.1. Where applicable, FDR adjustment of *P* values was performed to account for multiple comparisons.

An analysis of all key data with study participants stratified by biological sex is provided in Supplementary Fig. 7.

### Reporting summary

Further information on research design is available in the Nature Portfolio Reporting Summary linked to this article.

## Online content

Any methods, additional references, Nature Portfolio reporting summaries, source data, extended data, supplementary information, acknowledgements, peer review information; details of author contributions and competing interests; and statements of data and code availability are available at 10.1038/s41591-023-02243-5.

### Supplementary information

Supplementary Information Supplementary Table 1 and Supplementary Figs. 1–7.

Reporting Summary

Supplementary Table Supplementary Tables 2–17.

Supplementary Table 18 STORMS checklist.

## Figures and Tables

**Extended Data Table 1:**
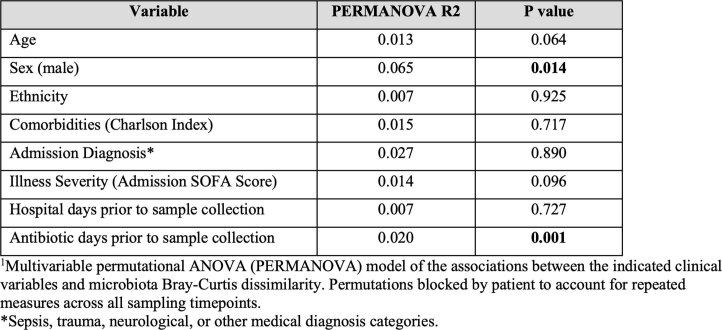
Multivariable analysis of clinical factors and their association with microbiota composition during the first week of ICU admission

**Extended Data Table 2:**
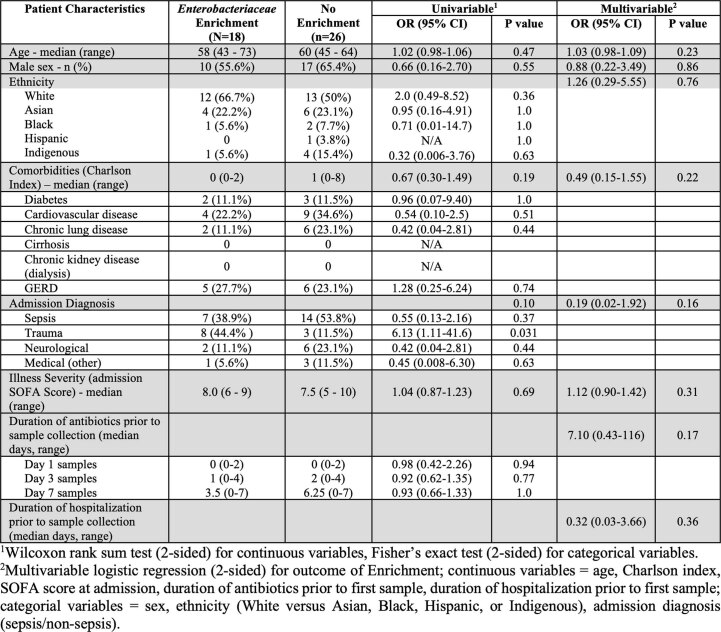
Demographic and clinical characteristics of patients with and without progressive *Enterobacteriaceae* enrichment in the fecal microbiota

## Data Availability

Correspondence and requests should be addressed to B.M. (bamcdona@ucalgary.ca). DNA sequence datasets have been deposited and are available in the NCBI Sequence Read Archive under BioProject ID PRJNA851469. Additional datasets are available in Supplementary Tables. Other de-identified datasets are available upon request. Access to metadata containing potentially identifying patient information requires an approved research ethics protocol and may require approval from Alberta Health Services as the steward of patient information for all study participants; a material/data transfer agreement may be required. A publicly available dataset or 16s rRNA gene sequences from Dadkhah et al.^22^ was used in this study, as well as the DADA2 formatted SILVA database v.138.1, which is available at 10.5281/zenodo.4587955.

## Code availability

All analyses were performed using publicly available software and published code as described in Methods. No custom code was generated for data analyses in this study.
