# Supplementary material for: Dysbiosis of a microbiota–immune metasystem in critical illness is associated with nosocomial infections
Source: Nat Med. 2023 Mar 9;29(4):1017–27. doi: 10.1038/s41591-023-02243-5 (PMC10115642; doi:10.1038/s41591-023-02243-5)
Supplement: Supplementary file 1 — Supplementary Table 1 and Supplementary Figs. 1–7. [file 41591_2023_2243_MOESM1_ESM.pdf]

nature medicine

Article

https://doi.org/10.1038/s41591-023-02243-5

Dysbiosis of a microbiota–immune metasystem in critical illness is associated with nosocomial infections

In the format provided by the authors and unedited

Supplementary Table 1 – Study patient timelines.

ID	Admission Diagnosis Category	Timeline of Sample Collection and Antibiotics	Nosocomial Infection to day 30	Mortality to day 30	Progressive *Enterobacteriaceae* enrichment
1	Neuro	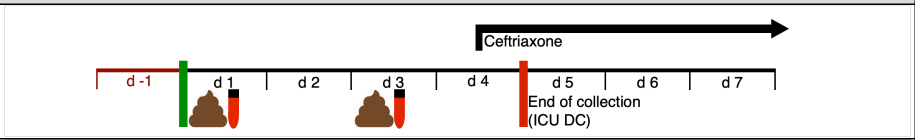	Yes (day 4)	No	No
2	Sepsis (CAP[1])	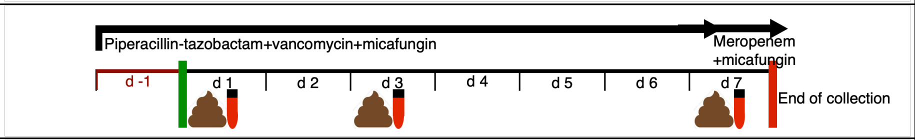	Yes (day 6)	No	No
3	Trauma	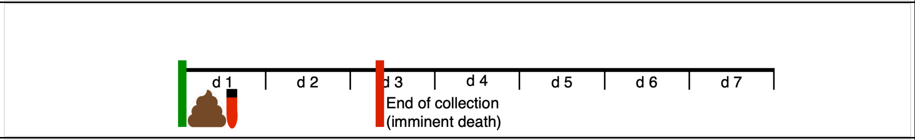	No	Yes (day 5)	n/a
4	Trauma	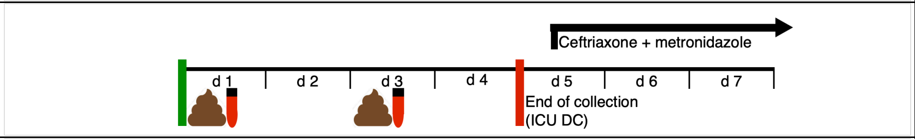	Yes (day 5)	No	Yes
5	Sepsis (CAP)	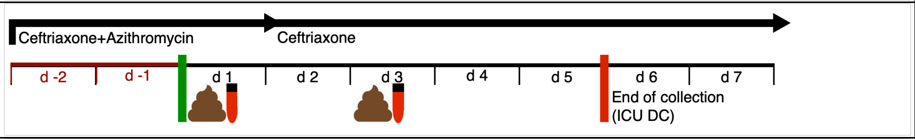	No	No	No
6	Neuro	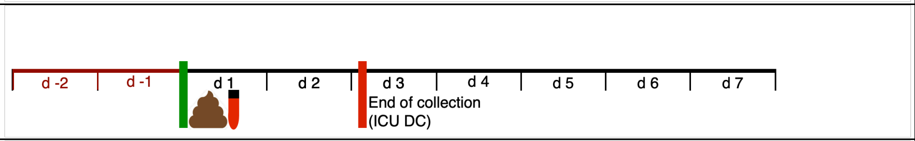	No	No	n/a
7	Sepsis (CAP)	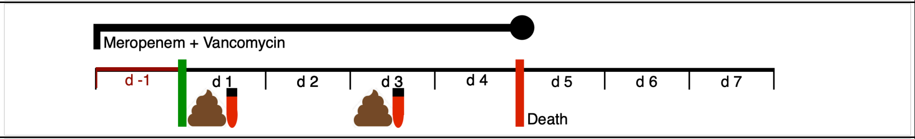	No	Yes (day 4)	No
8	Trauma	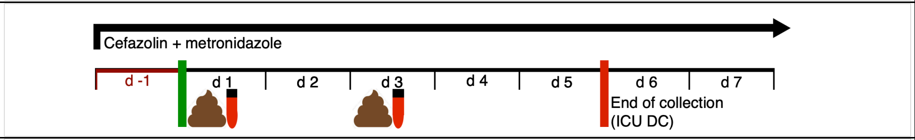	No	No	Yes
9	Trauma	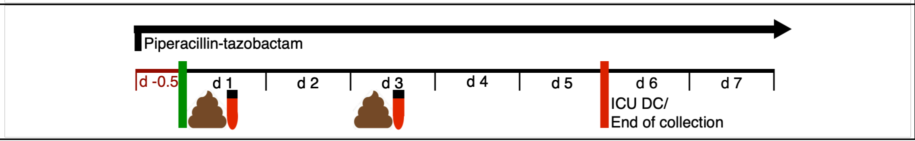	Yes (day 10)	Yes (day 11)	Yes

10	Neuro	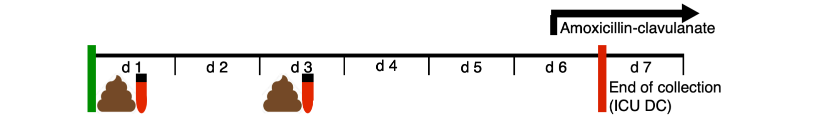	Yes (day 6)	No	No
11	Neuro	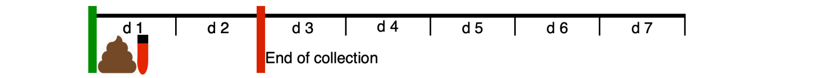	Yes (day 8)	No	n/a
12	Neuro	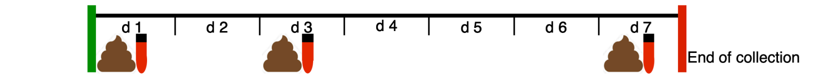	Yes (day 15)	No	No
13	Trauma	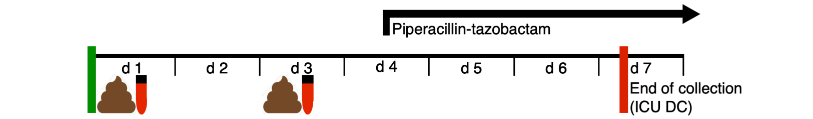	Yes (day 4)	No	Yes
14	Trauma	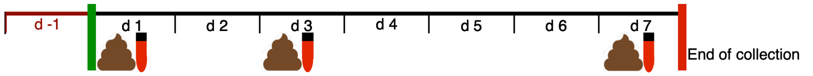	Yes (day 9)	Yes (day 24)	Yes
15	Sepsis (Pyelo-nephritis)	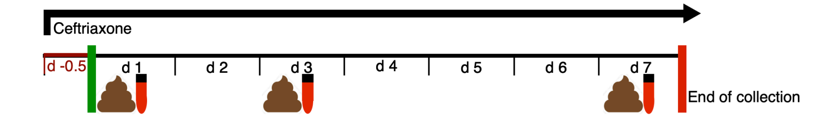	No	No	No
16	Sepsis (CAP)	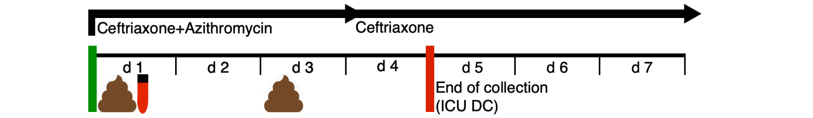	No	No	No
17	Sepsis (CAP)	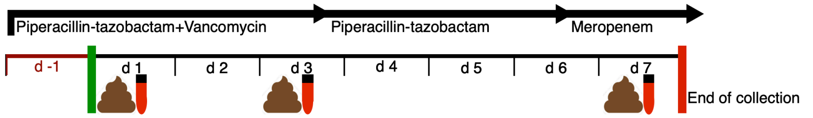	Yes (day 7)	No	Yes
18	Sepsis (CAP)	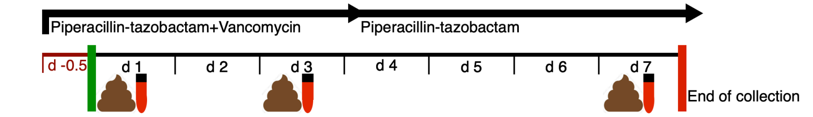	Yes (day 10)	No	No
19	Sepsis (CAP)	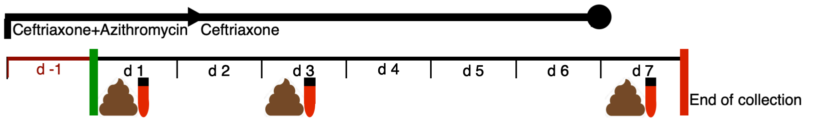	No	No	No

20	Trauma	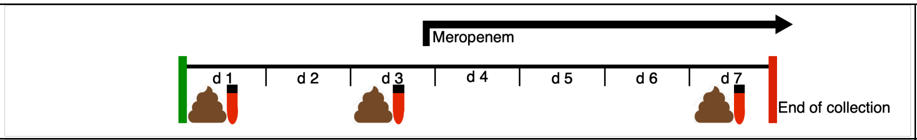	Yes (day 3)	No	Yes
21	Neuro	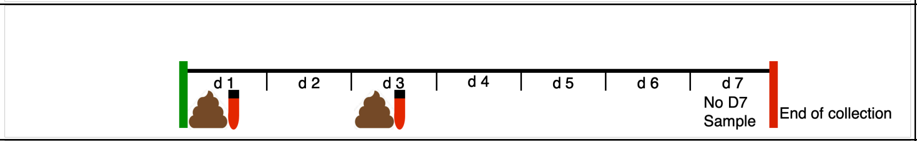	Yes (day 23)	No	No
22	Trauma	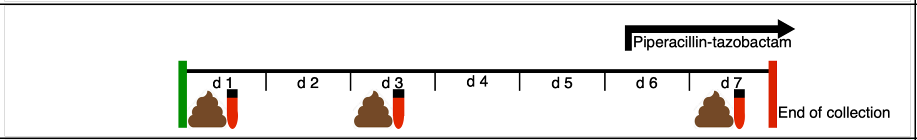	Yes (day 6)	No	No
23	Sepsis (IAI[2])	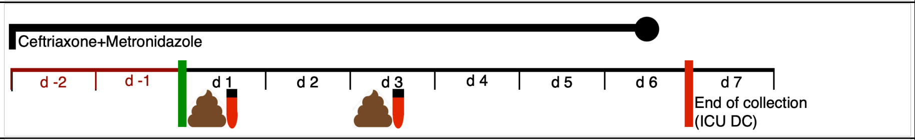	No	No	No
24	Sepsis (CAP)	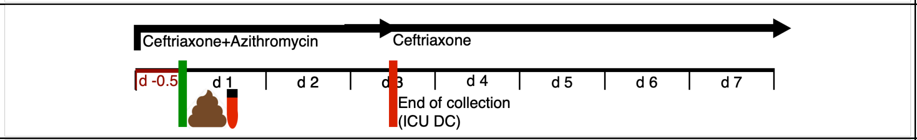	No	No	n/a
25	Sepsis (SSTI[3])	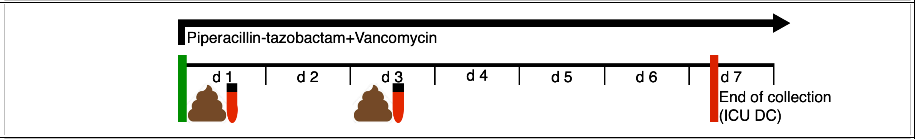	No	No	No
26	Sepsis (Bacter-emia)	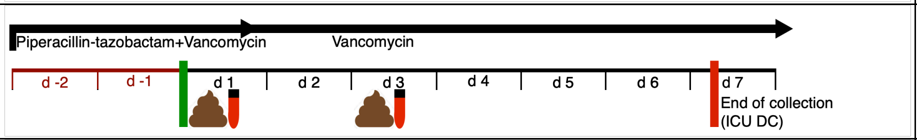	No	No	No
27	Sepsis (CAP)	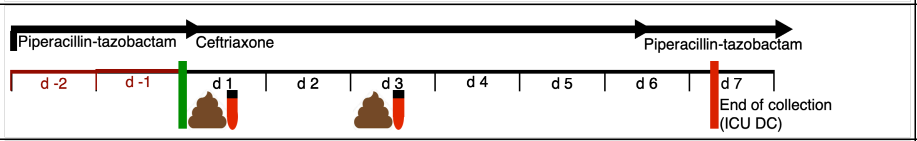	Yes (day 6)	No	Yes
28	Sepsis (Biliary)	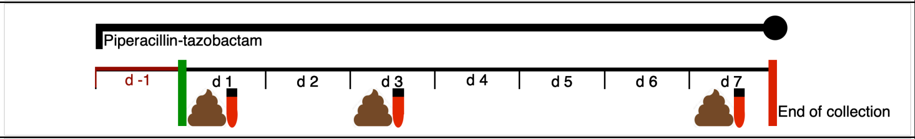	Yes (day 14)	Yes (day 26)	Yes
29	Medical (PE[4])	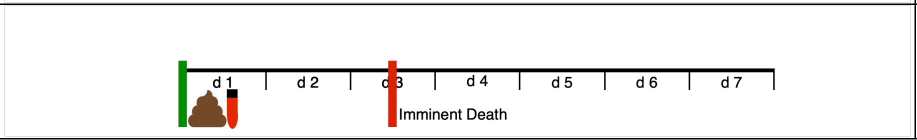	Yes (day 3)	Yes (day 4)	n/a

30	Sepsis (CAP)	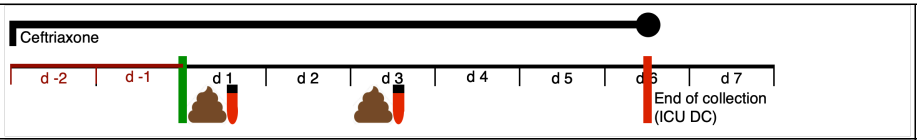	No	No	No
31	Sepsis (CAP)	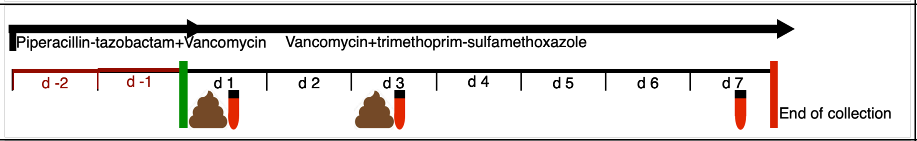	No	No	Yes
32	Medical (hemorrhage)	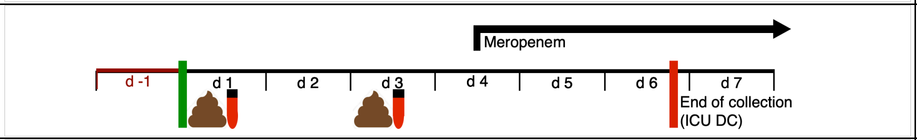	Yes (day 4)	Yes (day 15)	No
33	Sepsis (Sepsis NOS)	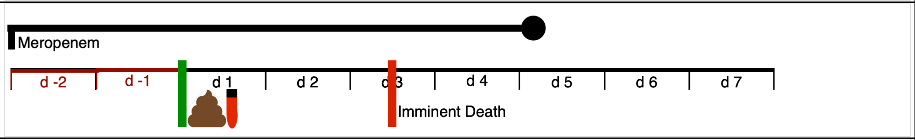	No	Yes (day 5)	n/a
34	Medical (cardiac arrest)	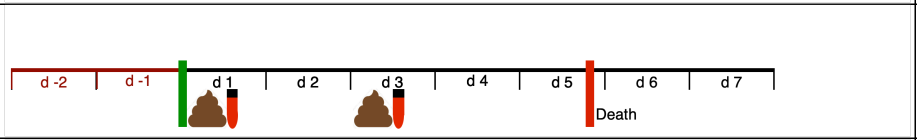	No	Yes (day 5)	No
35	Trauma	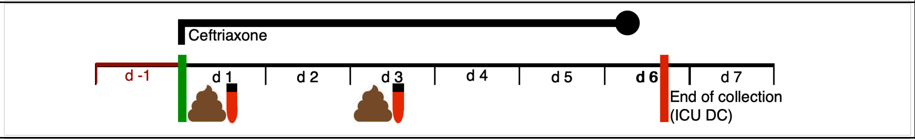	No	No	No
36	Medical (cardiac arrest)	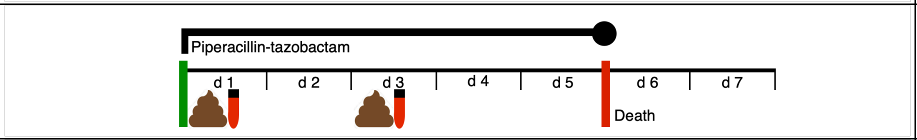	No	Yes (day 5)	No
37	Sepsis (IAI)	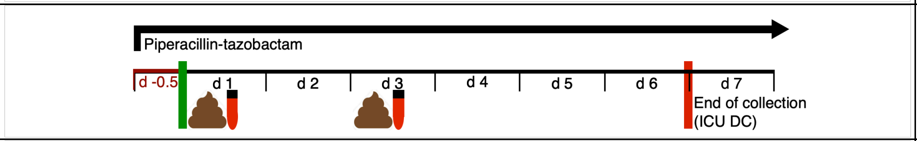	No	No	No
38	Sepsis (CAP)	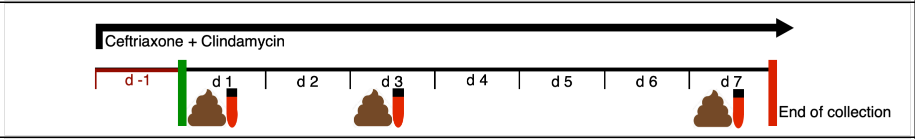	Yes (day 8)	No	Yes
39	Medical (cardiac arrest)	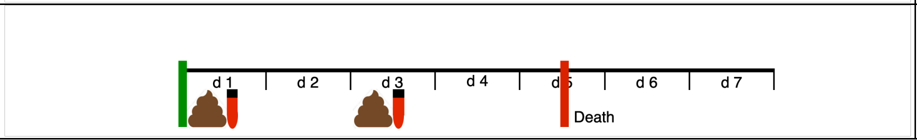	No	Yes (day 5)	Yes

40	Neuro	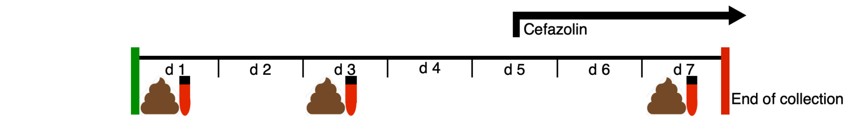	Yes (day 5)	No	No
41	Sepsis (CAP)	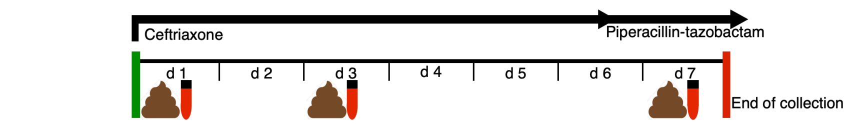	Yes (day 6)	No	No
42	Neuro	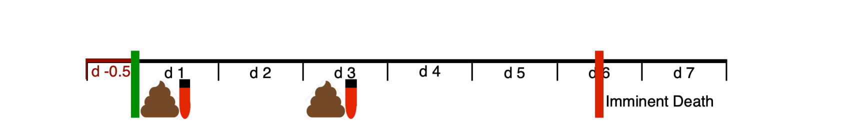	Yes (day 6)	Yes (day 9)	Yes
43	Sepsis (SSTI)	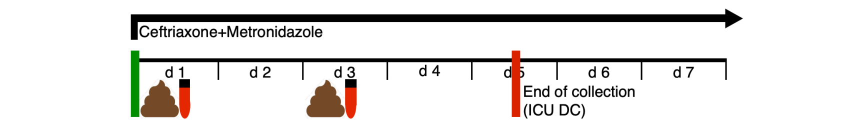	Yes (day 20)	No	Yes
44	Neuro	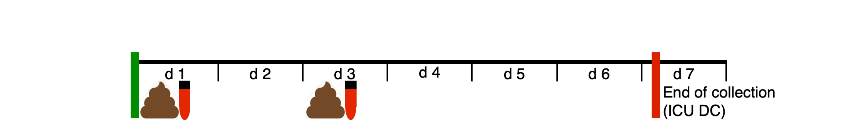	Yes (day 7)	Yes (day 17)	Yes
45	Trauma	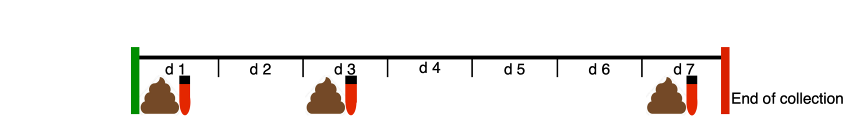	Yes (day 9)	No	Yes
46	Trauma	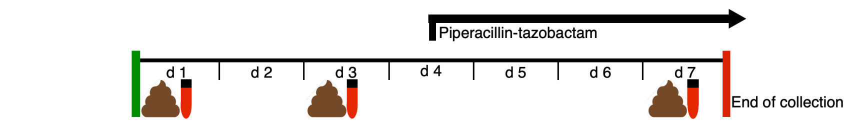	Yes (day 4)	No	Yes
47	Neuro	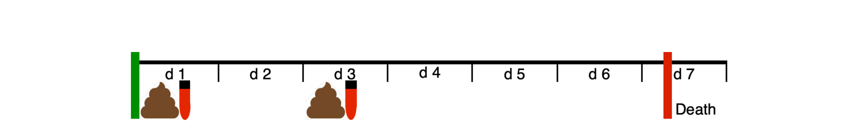	No	Yes (day 7)	No
48	Sepsis (CAP)	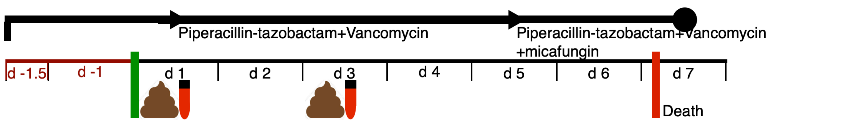	Yes (day 5)	Yes (day 7)	No
49	Trauma	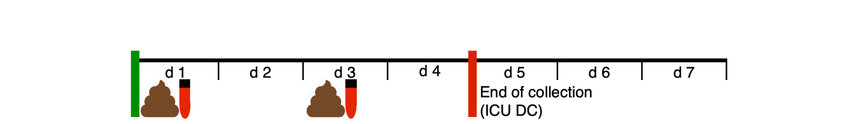	No	No	No

50	Sepsis (bacterial meningitis)	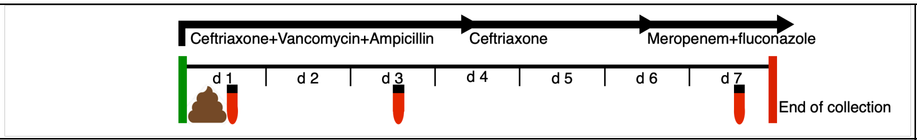	No	Yes (day 14)	n/a
51	Sepsis (CAP)	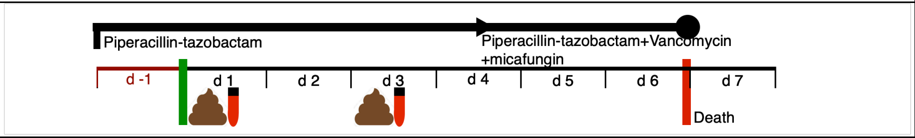	Yes (day 4)	Yes (day 6)	Yes

[1]CAP, community-acquired pneumonia, [2]IAI, intra-abdominal infection, [3]SSTI, skin and soft tissue infection, [4]PE, pulmonary thromboembolism

Supplementary Figure 1

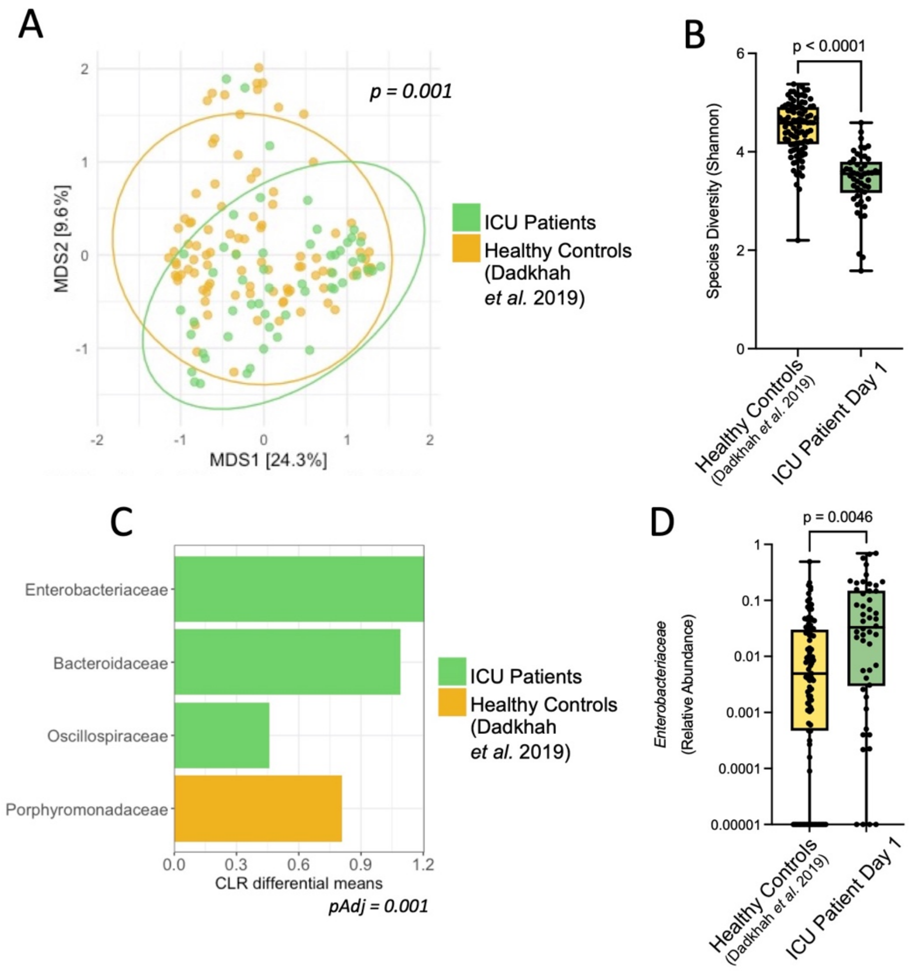

Supplementary Figure 1 – Fecal microbiota composition of ICU patients compared to age-matched healthy cohort dataset from Dadkhah *et al*.[23] **(A)** PCoA of fecal microbiota composition calculated on the Bray-Curtis dissimilarity distances at the ASV level, analyzed using a permutational analysis of variance (PERMANOVA). **(B)** Comparison of Shannon index between healthy controls and ICU patients on Day 1 of admission. Dots are individual patients, central line is median, box shows IQR, whiskers show range, statistical analysis by 2-sided Mann-Whitney test. P value as shown. **(C)** Differential abundance of bacterial families between ICU patients on Day 1 and healthy controls was determined using ANCOM-II, showing statistically significant families (Padj<0.1). **(D)** Relative abundance of *Enterobacteriaceae* in healthy controls compared to ICU patients on day 1 of admission. Dots are individual patients, central line is median, box shows IQR, whiskers show range, statistical analysis by Mann-Whitney test. P value as shown.

Supplementary Figure 2

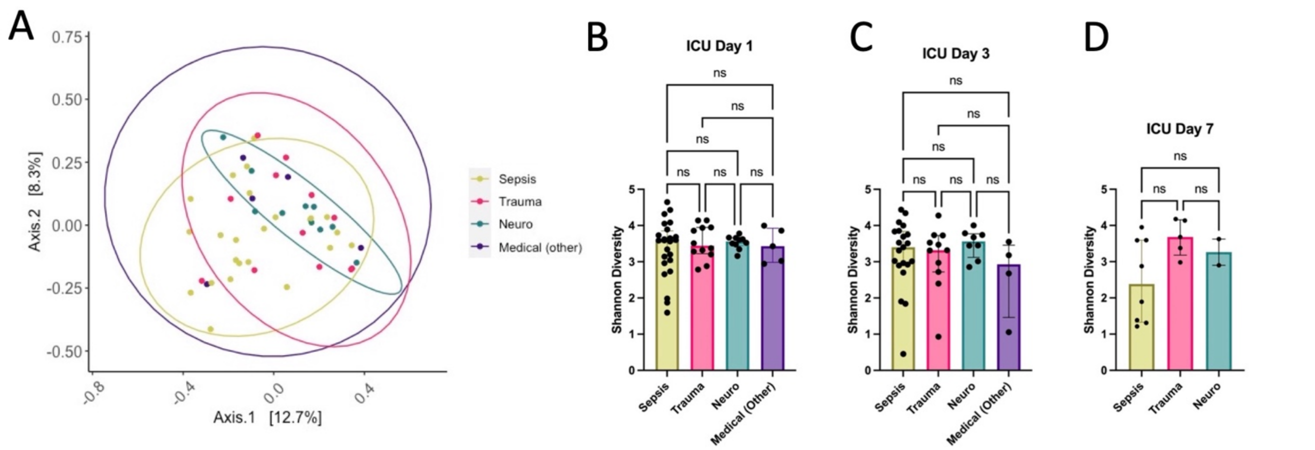

Supplementary Figure 2 – **Fecal microbiota in ICU patients with different admission diagnoses. (A)** PCoA of fecal microbiota composition calculated on the Bray-Curtis dissimilarity distances at the ASV level on Day 1 of ICU admission, analyzed using a permutational analysis of variance (PERMANOVA) between admission diagnoses. **(B,C,D)** Comparison of Shannon diversity between admission diagnoses on Days 1 (N=51), 3 (N=44), and 7 (N=15) of ICU admission. Dots are individual patients, and bars show median values, error bars show interquartile range. Statistical comparisons were performed using a Kruskal-Wallis test with post-hoc Tukey's test. ns = non-significant.

Supplementary Figure 3

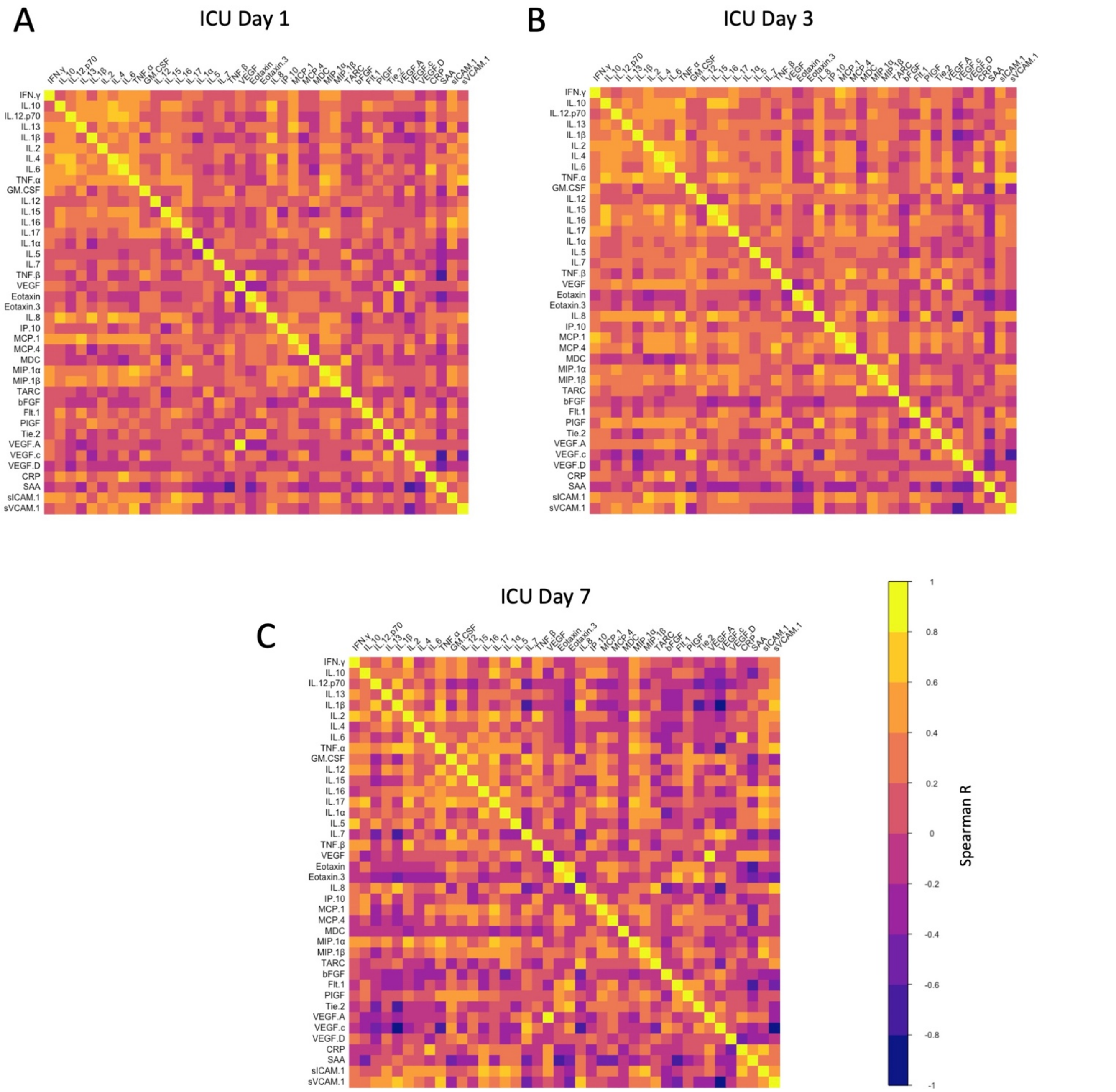

Supplementary Figure 3 – Systemic inflammatory response networks during critical illness. Correlation matrices of 40 inflammatory mediators in plasma collected from critically ill patients (N=51) on **(A)** day 1 of ICU admission (N=51), and again from survivors who remained in the ICU on **(B)** day 3 (N=43), and **(D)** day 7 (N=15). Heatmaps show Spearman's correlation coefficients.

Supplementary Figure 4

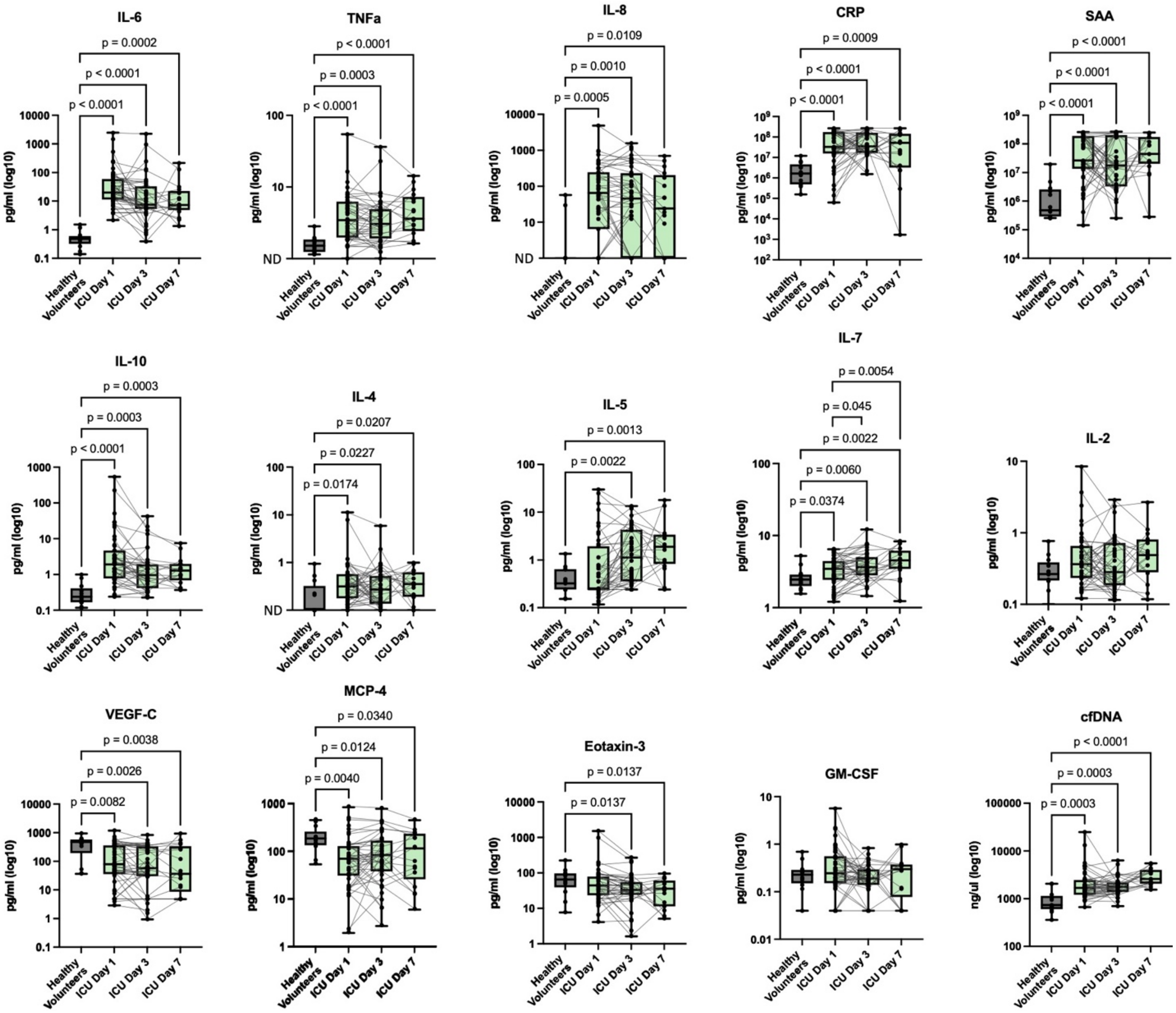

Supplementary Figure 4 – Systemic inflammatory response dynamics in critically ill patients. Quantities of 15 key inflammatory mediators measured in plasma samples from critically ill patients (N=51) sampled on day 1 of admission (N=51), and again from survivors who remined in ICU on day 3 (N=43), and day 7 (N=15), compared to healthy volunteer controls (N=13). Dots are individual patients, central line is median, box shows IQR, whiskers show range, statistical analysis by Mann-Whitney test Statistical comparisons between healthy volunteers and ICU patients at each timepoint were performed using a Kruskal-Wallis test, while pairwise comparisons of repeated measures across ICU patient-days were performed using mixed linear regression model with post-hoc Tukey's tests. P values as shown.

Supplementary Figure 5

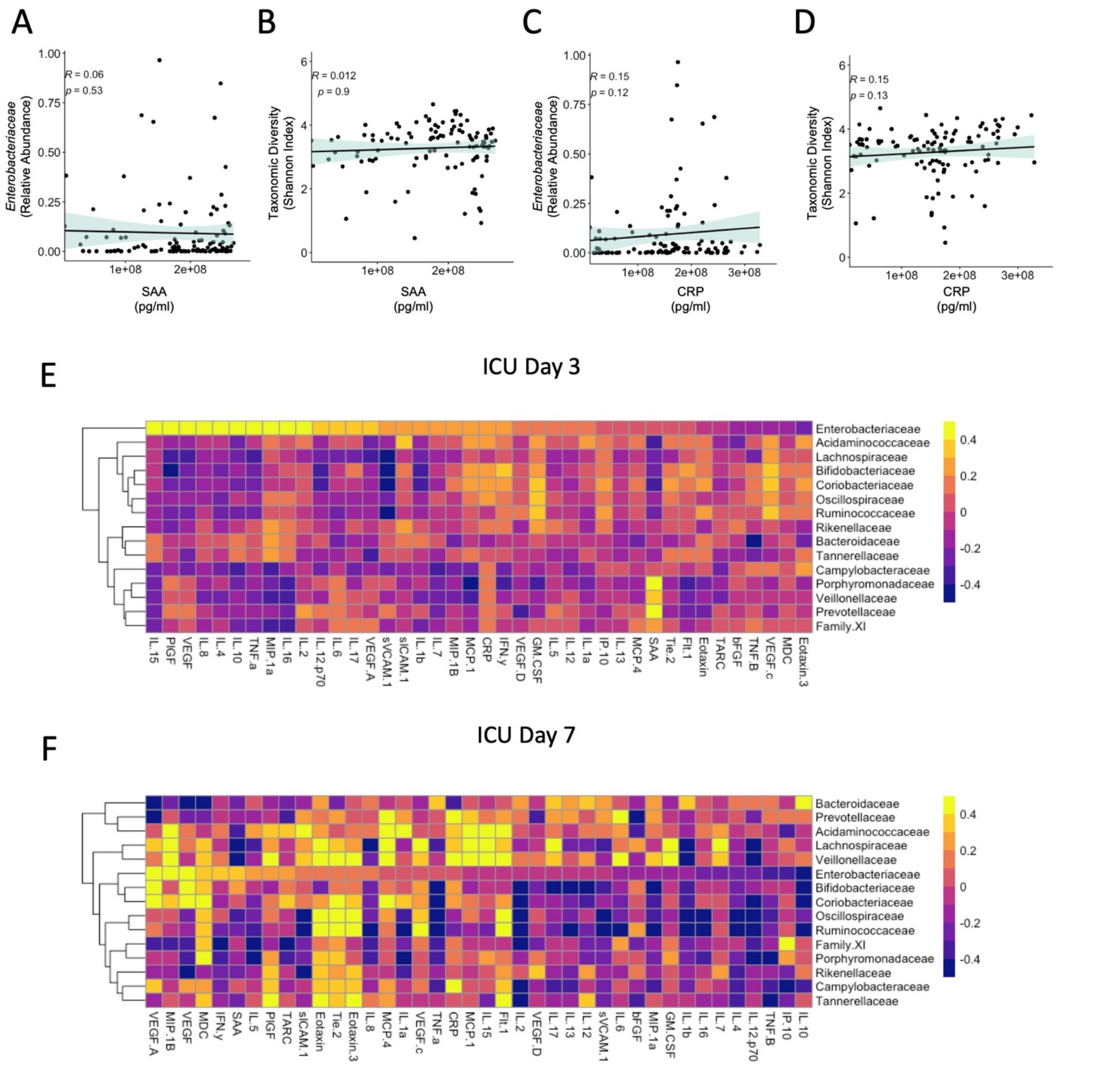

Supplementary Figure 5 – Correlations between fecal microbiota composition and systemic inflammatory response during critical illness. (A,B) Spearman correlations between plasma levels of SAA and (A) the relative abundance of *Enterobacteriaceae* and (B) microbiota diversity (Shannon index) in ICU patients across all sampling timepoints**. (C,D)** Spearman correlation between plasma concentration of CRP and (C) the relative abundance of Enterobacteriaceae and (D) microbiota diversity (Shannon index) in ICU patients across all sampling timepoints**.** Dots show individual patient samples (N=109 samples), regression (line) and 95% confidence intervals (shaded area) are shown. **(E,F)** Spearman's correlation coefficients were calculated between the 15 most abundant taxa in the fecal microbiota and the levels of inflammatory mediators measured in plasma collected from critically ill patients on **(A)** ICU day 3 (N=43), and **(B)** day 7 (N=15). Heatmaps show Spearman's correlation coefficients.

Supplementary Figure 6

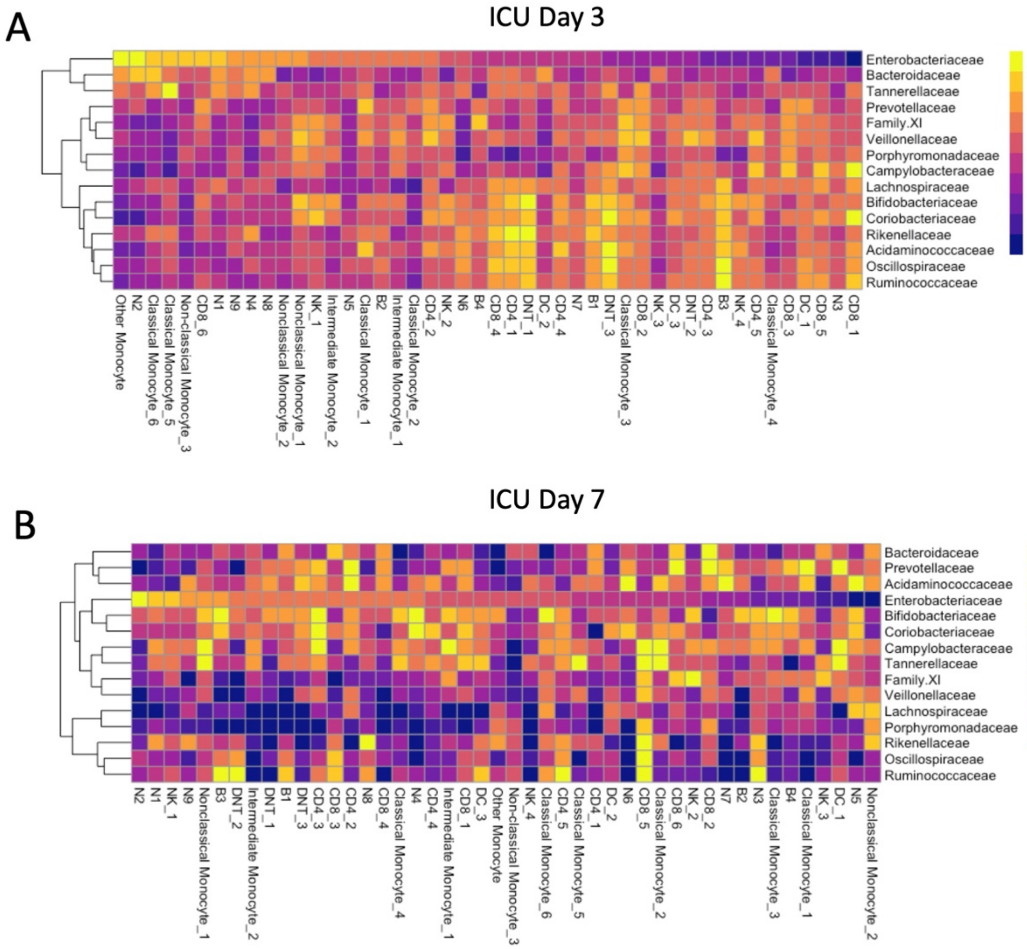

Supplementary Figure 6 – Correlations between fecal microbiota composition and cellular immune response during critical illness. Spearman's correlation coefficients were calculated between the 15 most abundant taxa in the fecal microbiota and the quantity of all innate and adaptive immune cell populations identified by unsupervised clustering of single cell mass cytometry data collected from critically ill patients on **(A)** day 3 of ICU admission (N=43) and **(B)** day 7 (N=15). Heatmaps show Spearman's correlation coefficients.

Supplementary Figure 7

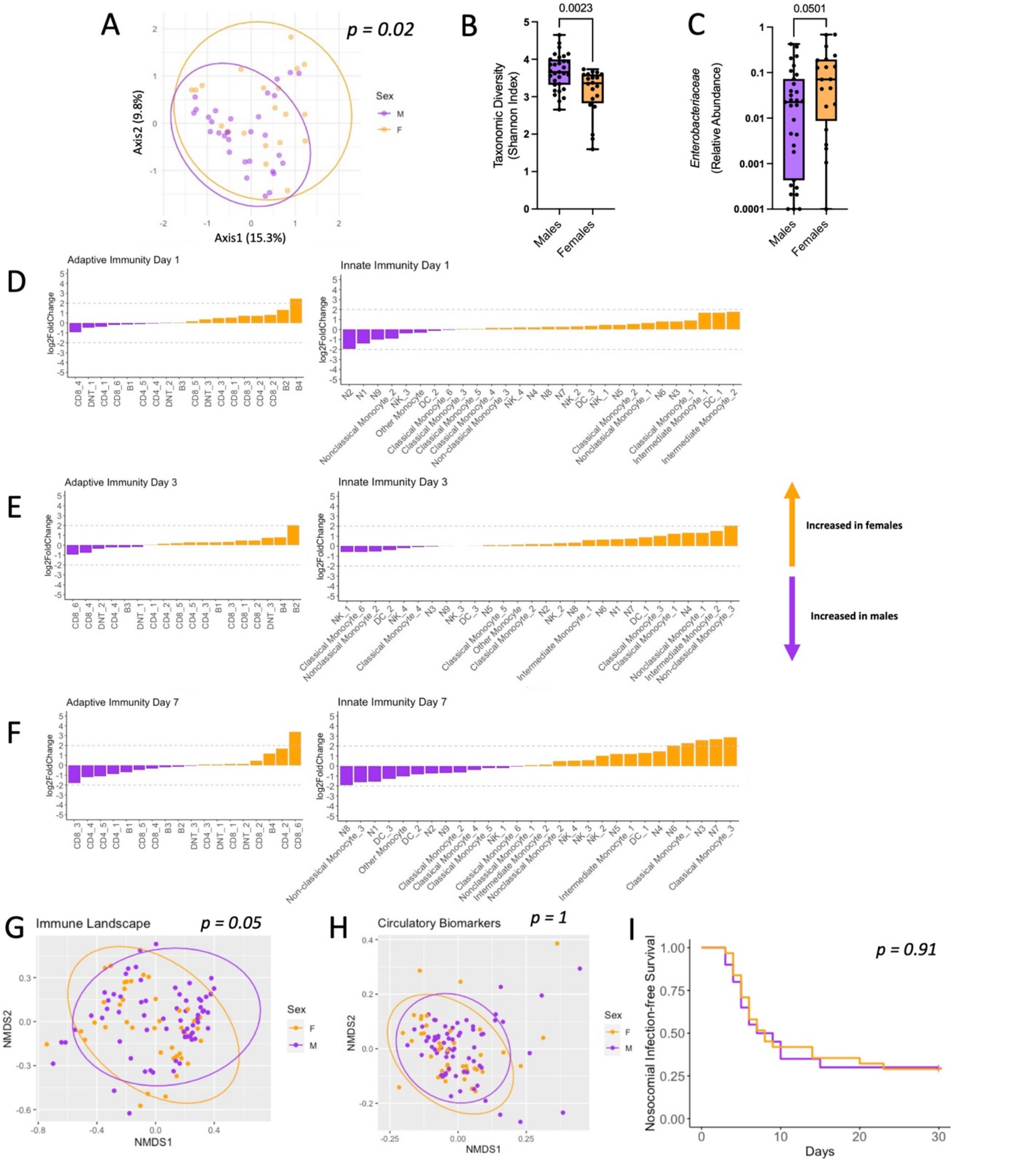

Supplementary Figure 7 – Sex disaggregated analyses of multi-omic datasets and outcomes of critically ill patients. (A) PCoA of fecal microbiota composition calculated on the Bray-Curtis dissimilarity distances at the ASV level, analyzed by PERMANOVA, **(B)** shannon index, and **(C)** relative abundance of *Enterobacteriaceae* between male (N=31) and female (N=20) ICU patients on Day 1. Dots are individual patients, central line is median, box shows IQR, whiskers show range, compared using a Mann-Whitney test. **(D,E,F)** Differential abundance analysis of cell counts of adaptive and innate immune cell clusters in blood on Days 1, 3, and 7 of ICU admission identified by clustering of mass cytometry data between male and female ICU patients. **(G,H)** NMDS ordination of (G) immune cell landscape in blood, and (H) the systemic inflammatory mediator landscape in plasma across all sampling timepoints in male and female ICU patients. Statistical comparisons were performed using PERMANOVA, each point is an individual patient-time point. To determine the independent impact of biological sex, analyses in A and D-H were controlled for clinical co-variables that were independently associated with immune cell composition (Supp Table 15). (I) Kaplan-Meier curve showing 30-day nosocomial infection-free survival between males and females, analyzed by long-rank test. *p* values as shown.
