# Supplementary material for: Dysbiosis of a microbiota–immune metasystem in critical illness is associated with nosocomial infections
Source: Nat Med. 2023 Mar 9;29(4):1017–27. doi: 10.1038/s41591-023-02243-5 (PMC10115642; doi:10.1038/s41591-023-02243-5)
Supplement: Supplementary file 2 — Reporting Summary [file 41591_2023_2243_MOESM2_ESM.pdf]

nature portfolio

Corresponding author(s): Braedon McDonald

Last updated by author(s): Feb 21, 2023

Reporting Summary

Nature Portfolio wishes to improve the reproducibility of the work that we publish. This form provides structure for consistency and transparency in reporting. For further information on Nature Portfolio policies, see our Editorial Policies and the Editorial Policy Checklist.

Statistics

For all statistical analyses, confirm that the following items are present in the figure legend, table legend, main text, or Methods section.

n/a	Confirmed	
☐	☒	The exact sample size (*n*) for each experimental group/condition, given as a discrete number and unit of measurement
☐	☒	A statement on whether measurements were taken from distinct samples or whether the same sample was measured repeatedly
☐	☒	The statistical test(s) used AND whether they are one- or two-sided<br>*Only common tests should be described solely by name; describe more complex techniques in the Methods section.*
☐	☒	A description of all covariates tested
☐	☒	A description of any assumptions or corrections, such as tests of normality and adjustment for multiple comparisons
☐	☒	A full description of the statistical parameters including central tendency (e.g. means) or other basic estimates (e.g. regression coefficient) AND variation (e.g. standard deviation) or associated estimates of uncertainty (e.g. confidence intervals)
☐	☒	For null hypothesis testing, the test statistic (e.g. *F*, *t*, *r*) with confidence intervals, effect sizes, degrees of freedom and *P* value noted<br>*Give P values as exact values whenever suitable.*
☒	☐	For Bayesian analysis, information on the choice of priors and Markov chain Monte Carlo settings
☒	☐	For hierarchical and complex designs, identification of the appropriate level for tests and full reporting of outcomes
☐	☒	Estimates of effect sizes (e.g. Cohen's *d*, Pearson's *r*), indicating how they were calculated

Our web collection on statistics for biologists contains articles on many of the points above.

Software and code

Policy information about availability of computer code

Data collection	16S rRNA gene amplification and sequencing: Rectal swabs were collected and stored in sterile tubes at -80 ºC. DNA was isolated using the DNeasy PowerSoil (Qiagen) following the manufacturer's protocol. PCR amplification of the 16S V4 region was performed using previously described dual indexed primers with sample barcodes and sequencing adaptors and PCR conditions 44. PCR products were cleaned and size selected using Nucleomag beads (Macherey Nagel) following manufacturer's instructions. Individual sample libraries were normalized using a SequalPrep Normalization Plate (Invitrogen), after which samples were pooled to create the final library. Quality control of the pooled NGS library was performed using an Agilent Technologies 2200 TapeStation and Qubit dsDNA analyzer. The pooled 16S V4 amplicon library was sequenced using an Illumina MiSeq platform to produce 2 x 250bp paired-end reads.<br><br>Time-of-flight mass cytometry: Whole blood samples used for mass cytometry analysis were cryopreserved in PROT1 proteomic stabilizer (SmartTube) at a ratio of 1:1.4 and stored at -80ºC to enable batched analysis of patient samples. Samples were thawed at room temperature, and RBC lysis was performed using PROT1 RBC lysis buffer (SmartTube), and white blood cells were washed in cell staining medium (PBS with 1% BSA) followed by labelling with a custom metal-conjugated antibody panel (see Extended Data Table 4). White blood cells were incubated with metal-conjugated surface antibodies, followed by fixation and permeabilized (BD Cytofix-Cytoperm), incubation with intracellular antibodies, then left overnight in a solution containing Cell-ID iridium intercalator (Fluidigm), 0.3% saponin, and 1.6% paraformaldehyde in PBS. Cells were then mixed with EQ Four Element Calibration Beads (Fluidigm) and acquired on a Helios CyTOFII mass cytometer (DVS). Mass cytometry data was normalized using the internal Helios CyTOFII bead-based normalization software (DVS).
Data analysis	16S amplicon sequence data processing and analysis: De-multiplexed Illumina MiSeq paired-end reads (FASTQ) were processed in R v4.1.2 following the DADA2 pipeline v1.14. Forward and reverse reads were truncated to 230bp and 210bp, respectively, or to the first base with a quality score Q<2. Reads containing any ambiguous (N) nucleotides or reads containing more than 2 errors were removed. Samples with less than 1000 reads following trimming and filtering steps were discarded from further analysis. Taxonomy of unique ASVs was assigned in

DADA2 by the RDP Classifier using the SILVA v138.1 database. ASVs and sample data were combined using the Phyloseq package v.1.38.0 for further downstream analysis. Potential contaminants were identified and removed by the Decontam package v.1.14.0 based on the distributed frequency of ASVs and the DNA concentration of individual samples. ASVs taxonomically assigned to Cyanobacteria, mitochondria, or chloroplast were removed.

Microbiome alpha-diversity metrics were calculated using the Microbiome package v.1.16.0. Community dissimilarity (beta-diversity) was calculated on the Bray-Curtis dissimilarity measure by permutational ANOVA (PERMANOVA) using the adonis function in Vegan v.2.6, and three-dimensional visualization was performed by plotting the first three dimensions of the Bray-Curtis dissimilarity ordination using the plotly package v.4.10.0. Spearman correlation matrices were performed between the 15 most abundant bacterial families present in at least 10% of samples using the rcorr function in the Hmisc package v.4.7 and visualized using the ggplot2 package v3.3.6 in R. Taxonomy plots showing relative abundances were plotted using ggplot2 package microbiomeutilities v.1.00.16 and the Phyloseq packages v1.38.0 in R. Penalized ridge regression analysis (glmnet R package v4.1-4) was used to determine the importance of the 15 most abundant bacterial families towards specified microbiome outcomes (change in Shannon diversity or change in Enterobacteriaceae relative abundance between ICU days 1 and 3) using a 3-fold cross validation repeated 10 times through the R package caret v.6.0-93. Results were visualized using the vip package v.0.3.2 in R. Community stability was determined using the codyn R package on data from Days 1 and 3 of ICU admission. Differential abundance analysis was performed using ANCOM-II on relative abundances, with patients fit as a random effect to account for repeat measures using the microbiomeMarker R package v.1.0.2. Network analysis was performed on bacterial families using the NetCoMi R package65 v1.1.0.

Single cell mass cytometry data processing and analysis: Normalized mass cytometry data files were further processed in R using the CytoSpill package v.0.1.0 to correct for any signal overlap between markers. Next, corrected FCS files were imported into Cytobank (Cytobank, Inc) for manual gating on CD45+ single-cell events and major cell populations (Supplementary Table 8). Manually gated events were then exported as FCS files for further analysis in R using the CATALYST package v.1.16.0. Batch correction was performed using the RemoveBatchEffect function in the limma package v.3.48.3. Gated cell populations were clustered based on the expression of all available markers in CATALYST using the FlowSOM function. Extremely rare metaclusters (less 0.5% of events in each population) or aberrant clusters (aberrant expression of all panel markers or less than three panel markers) were also removed. tSNE dimensionality reduction was performed on 1000 randomly selected events from each sample using a perplexity of 80 for 5000 iterations. Figures were generated within the built-in functions of CATALYST in R. Visualization of dimensionality-reduced cellular immune landscapes between study subjects was performed using non-metric multidimensional scaling (NMDS) of the relative abundance of immune cell populations using the Vegan metaMDS function in R, with statistical analysis using a permutational ANOVA (PERMANOVA) in the Vegan R Vegan package v.2.6. Figures showing log2 fold change of individual cell populations (between healthy volunteers and ICU patients, or between ICU patients with or without Enterobacteriaceae enrichment) were calculated on absolute cell counts and controlled for clinical covariables that were significantly associated with immune cell composition (Supplementary Table 15) using the DESeq2 package v.1.34.0.

Multi-omics integration and analysis: To perform an integrated multi-omics analysis of the fecal microbiota, cellular immune composition, and inflammatory mediator landscape in blood, we employed an unsupervised factor analysis approach in R with the Multi-Omics Factor Analysis (MOFA) package v.1.4.0. Briefly, microbiota taxonomic data was aggregated to the family level and filtered to a cutoff of 25% prevalence, after which the count data was transformed by center log ratio (clr). Single-cell mass cytometry count data and inflammatory mediator concentrations were log transformed prior to dataset integration. The resulting MOFA factors were then compared between healthy volunteers and ICU patients to determine latent factors that explain variation between these populations. Modelling of the combined datasets in MOFA was performed using the default parameters. Briefly, model fitting identified the top 10 MOFA factors that explained the largest amount of variation between samples. Next, MOFA factors showing contributions from all meta-systems datasets (fecal microbiota, single cell immune composition, systemic inflammatory mediators) that explained at least 5% of variance were compared between healthy volunteers and ICU patients. Within each MOFA factor, individual features weights (eg. individual microbial taxa within microbiota factors) were compared between healthy volunteers and ICU patients.

Connectivity between microbiota, cellular immune landscape, and inflammatory mediators was determined using Chord diagram analysis. A Spearman correlation coefficient was calculated for pairings of all microbial taxa (relative abundance, family level), immune cell subset counts, and inflammatory mediatory concentrations (FDR-adjusted for multiple comparisons with $p < 0.1$), and significant values were visualized using the circlize package v.0.4.15 in R. Heatmaps depicting the Spearman correlation coefficients between the 15 most abundant bacterial families and immune components (cell counts and inflammatory mediator concentrations) were generated using the rcorr function in the Hmisc package v.4.7, and visualized using the pheatmap package v.1.0.12 in R. Differential abundance of 40-plex plasma inflammatory biomarkers was performed on the log2 transformed concentration values using the limma package v.3.48.3 in R.

For manuscripts utilizing custom algorithms or software that are central to the research but not yet described in published literature, software must be made available to editors and reviewers. We strongly encourage code deposition in a community repository (e.g. GitHub). See the Nature Portfolio guidelines for submitting code & software for further information.

Data

Policy information about availability of data

All manuscripts must include a data availability statement. This statement should provide the following information, where applicable:

- Accession codes, unique identifiers, or web links for publicly available datasets
- A description of any restrictions on data availability
- For clinical datasets or third party data, please ensure that the statement adheres to our policy

Author to whom correspondence and requests should be addressed: Braedon McDonald (bamcdona@ucalgary.ca). DNA sequence datasets have been deposited and are available in the NCBI Sequence Read Archive under BioProject ID PRJNA851469. Additional datasets are available in Supplementary Tables. Other de-identified datasets are available upon request. Access to metadata containing potentially identifying patient information require an approved research ethics protocol and may require approval from Alberta Health Services (AHS) as the steward of patient information for all study subjects, and a material/data transfer agreement may be required. A publicly available dataset or 16s rRNA gene sequences from Dadkhah et al.23 was used in this study, as well as the DADA2 formatted SILVA database v138.1 which is available at DOI 10.5281/zenodo.4587955.

Human research participants

Policy information about studies involving human research participants and Sex and Gender in Research.

Reporting on sex and gender	A comprehensive analysis of primary data disaggregated for biological sex is provided in Supplementary Fig 7, with additional data on biological sex contributions in Extended Data Table 1, 2, and Supplementary Tables 15 and 16. Biological sex data for study cohorts is provided in Table 1.
Population characteristics	Study participant information (demographics, clinical characteristics, outcomes) are provided in Table 1 and timelines in Supplementary Table 1.<br><br>Study inclusion criteria included: adult (>18 years of age) with an index admission to ICU, requiring mechanical ventilation, who was expected to require continuous mechanical ventilation for >72 hours as judged by the treating ICU specialist. Study exclusion criteria were: pre-existing immunocompromised state (systemic immunomodulatory therapy, chemotherapy, HIV infection, other congenital or acquired immunodeficiency), had been hospitalized >48h prior to ICU admission in the previous 3 months, had received systemic anti-microbial therapy in the previous 3 months, had inflammatory bowel disease or active GI malignancy, prior surgery leaving a discontinuous GI tract, pregnancy, goals of care that excluded life-support interventions, or moribund patients not expected to survive >72 hours. At the onset of the COVID-19 pandemic, the study team added SARS-CoV-2 infection as an exclusion criteria, and therefore no patients with COVID-19 were included in this study.
Recruitment	Enrollment occurred between July 2019 and July 2021, with substantial delays and disruptions in enrollment due to the COVID-19 pandemic between March 2020-April 2021. Patients admitted to the medical, surgical, neurological, and trauma ICUs at the Foothills Medical Centre in Calgary, AB, Canada were screened daily for inclusion and exclusion criteria. For those meeting study criteria who agreed to participate, written informed consent was obtained from all study participants or appropriate surrogate decision maker for patients who were unable to provide consent due to incapacitating illness.
Ethics oversight	This study was approved by the conjoint health research ethics board of the University of Calgary and Alberta Health Services (REB18-1294). Written informed consent was obtained from all study participants or appropriate surrogate decision maker for patients who were unable to provide consent due to incapacitating illness.

Note that full information on the approval of the study protocol must also be provided in the manuscript.

Field-specific reporting

Please select the one below that is the best fit for your research. If you are not sure, read the appropriate sections before making your selection.

☒ Life sciences ☐ Behavioural & social sciences ☐ Ecological, evolutionary & environmental sciences

For a reference copy of the document with all sections, see nature.com/documents/nr-reporting-summary-flat.pdf

Life sciences study design

All studies must disclose on these points even when the disclosure is negative.

Sample size	This study enrolled 51 critically ill patients admitted to ICU. There were no previously published point estimates for multi-omics microbiome-immune outcomes for use in the calculation of a formal sample size estimate. Previous point prevalence studies found ICU acquired infections occurring in 22% of critically ill patients (Vincent JL, JAMA, 2017). However, as a discovery-based study of microbiota-immune interactions, there were no previously published effect size estimate to facility an a priori sample size calculation, therefore we enrolled a cohort size that was comparable to other recently published multi-omic studies in critically ill patients, as well as human microbiota-immune omics studies.Therefore, our exploratory analysis protocol aimed to enroll 50 ICU patients. 18 healthy controls will we included as comparator.
Data exclusions	1 enrolled patient was excluded from the study because the patients goals of care changed to end-of-life care and withdrawal of life sustaining interventions shortly 1 hour after enrollment on the day of admission due to an unexpected medical emergency. As above, goals of care excluding life sustaining interventions were an a priori exclusion criteria for this study. Given that the development of an exclusion criteria occurred approximately concurrently with enrollment, this patient was excluded and samples were not collected.
Replication	Experiments were conducted in batches between May 2019 and December 2021 to confirm that data were reproducible over time and between batches. Patient were included in individual batches. For mass cytometry data, slight batch effect in signal intensities were noted and therefore batch correction was performed using the RemoveBatchEffect function in the limma package v.3.48.3 in R.
Randomization	In this study, separation of ICU patients into groups based on microbiota characteristics (those with and without fecal Enterobacteriaceae enrichment) was performed retrospectively after sample collection, sequencing, and analysis. This could not be determined a priori, and therefore randomized allocation was not possible.
Blinding	Experiments in this study were performed by personnel who were blinded to patient status.

Reporting for specific materials, systems and methods

We require information from authors about some types of materials, experimental systems and methods used in many studies. Here, indicate whether each material, system or method listed is relevant to your study. If you are not sure if a list item applies to your research, read the appropriate section before selecting a response.

Materials & experimental systems

n/a	Involved in the study
☐	☒ Antibodies
☒	☐ Eukaryotic cell lines
☒	☐ Palaeontology and archaeology
☒	☐ Animals and other organisms
☐	☒ Clinical data
☒	☐ Dual use research of concern

Methods

n/a	Involved in the study
☒	☐ ChIP-seq
☒	☐ Flow cytometry
☒	☐ MRI-based neuroimaging

Antibodies

Antibodies used: All antibodies, clone, staining dilutions, supplier, and catalogue number are provided in Supplementary Table 17.

Validation: Metal conjugated antibodies from Fluidigm were validated by the supplier, who provided with the following validation statement on the technical data sheets, "Each lot of conjugated antibody is quality control tested by CyTOF (r) analysis of stained cells using the appropriate positive and negative cell staining and/or activation controls." In addition, we performed quality control and titration of labeling concentrations for each antibody in preliminary experiments.

Clinical data

Policy information about clinical studies

All manuscripts should comply with the ICMJE guidelines for publication of clinical research and a completed CONSORT checklist must be included with all submissions.

Clinical trial registration: This observational cohort study is not a clinical trial and is not registered with ClinicalTrials.gov.

Study protocol: Study protocol provided to the journal during submission, and is available from corresponding author upon request.

Data collection: Enrollment occurred between July 2019 and July 2021, with substantial delays and disruptions in enrollment due to the COVID-19 pandemic between March 2020-April 2021. Patients admitted to the medical, surgical, neurological, and trauma ICUs at the Foothills Medical Centre in Calgary, AB, Canada.

Outcomes:
Primary Clinical Outcomes:
1. Nosocomial infections occurring between admission/enrollment to day 30.
2. Mortality occurring between admission/enrolment to day 30.

Secondary clinical outcome measures:
1. Organ dysfunction: Sequential organ failure scores (SOFA components: GCS, PaO2:FiO2 ratio, blood pressure, renal function, liver function, and platelet count).
2. Duration of ICU admission and hospital admission.
